# Clonality and antigen-specific responses shape the prognostic effects of tumor-infiltrating T cells in ovarian cancer

**DOI:** 10.18632/oncotarget.27666

**Published:** 2020-07-07

**Authors:** Takemasa Tsuji, Kevin H. Eng, Junko Matsuzaki, Sebastiano Battaglia, J. Brian Szender, Anthony Miliotto, Sacha Gnjatic, Wiam Bshara, Carl D. Morrison, Shashikant Lele, Ryan O. Emerson, Jianmin Wang, Song Liu, Harlan Robins, Amit A. Lugade, Kunle Odunsi

**Affiliations:** ^1^Center for Immunotherapy, Roswell Park Comprehensive Cancer Center, Buffalo, NY, USA; ^2^Biostatistics and Bioinformatics, Roswell Park Comprehensive Cancer Center, Buffalo, NY, USA; ^3^Department of Cancer Genetics and Genomics, Roswell Park Comprehensive Cancer Center, Buffalo, NY, USA; ^4^Department of Gynecologic Oncology, Roswell Park Comprehensive Cancer Center, Buffalo, NY, USA; ^5^Tisch Cancer Institute, Icahn School of Medicine at Mount Sinai, New York, NY, USA; ^6^Department of Pathology, Roswell Park Comprehensive Cancer Center, Buffalo, NY, USA; ^7^Adaptive Biotechnologies, Seattle, WA, USA; ^*^These authors contributed equally to this work

**Keywords:** T-cell repertoire, ovarian cancer, tumor immunity

## Abstract

CD8^+^ tumor-infiltrating lymphocytes (TILs) are not all specific for tumor antigens, but can include bystander TILs that are specific for cancer-irrelevant epitopes, and it is unknown whether the T-cell repertoire affects prognosis. To delineate the complexity of anti-tumor T-cell responses, we utilized a computational method for *de novo* assembly of sequences from CDR3 regions of 369 high-grade serous ovarian cancers from TCGA, and then applied deep TCR-sequencing for analyses of paired tumor and peripheral blood specimens from an independent cohort of 99 ovarian cancer patients. Strongly monoclonal T-cell repertoires were associated with favorable prognosis (PFS, HR = 0.65, 0.50–0.84, *p* = 0.003; OS, HR = 0.61, 0.44–0.83, *p* = 0.006) in TCGA cohort. In the validation cohort, we discovered that patients with low T-cell infiltration but low diversity or focused repertoires had clinical outcomes almost indistinguishable from highly-infiltrated tumors (median 21.0 months versus 15.9 months, log-rank *p* = 0.945). We also found that the degree of divergence of the peripheral repertoire from the TIL repertoire, and the presence of detectable spontaneous anti-tumor immune responses are important determinants of clinical outcome. We conclude that the prognostic significance of TILs in ovarian cancer is dictated by T-cell clonality, degree of overlap with peripheral repertoire, and the presence of detectable spontaneous anti-tumor immune response in the patients. These immunological phenotypes defined by the TCR repertoire may provide useful insights for identifying “TIL-low” ovarian cancer patients that may respond to immunotherapy.

## INTRODUCTION

The presence of tumor-infiltrating lymphocytes (TILs) is a key determinant of clinical outcome in a wide range of solid tumors including ovarian cancer [1–5]. Although previous studies have identified additional parameters that associate with effective anti-tumor immunity, such as the differentiation state of TILs [2, 4], the ratio of effector to immunosuppressive cells [1, 5, 6], and tumor production of immune inhibitory molecules [7], these do not fully explain the varied and dynamic range of immune components influencing prognosis. The complex relationship between TILs, clinical prognosis, and response to immunotherapy is further complicated by recent studies indicating that TILs not only comprise of tumor antigen-specific cells, but also significant frequencies of T cells recognizing a wide range of epitopes irrelevant to cancer (e. g. influenza virus) [8]. In these studies, bystander CD8^+^ TILs were shown to lack CD39 expression [8], while tumor-specific T cells have been defined by expression of a variety markers including PD-1 [9], CD103 [10], CD39 [11] and CD137 [12]. While these markers are useful for differentiating tumor-reactive T cells within TILs, they do not address how the degree of clonal infiltration shapes the functional attributes of tumor-specific TILs and ultimately tumor control.

T lymphocytes express highly diverse antigen-specific receptors (TCR) created during T cell development by a unique process of somatic recombination, and up to 10^16^ TCR types have been reported [13]. The random addition and deletion of nucleotides during T cell receptor (TCR) recombination in the complementarity-determining region 3 (CDR3) provides variation in TCR sequences that is shaped further by selection and clonal expansion following exposure to immunogenic antigens. In this regard, characterizing the specific type and number of TCR clonotypes deployed to engage tumor antigens both at the tumor site and in the periphery may provide unique qualitative insights into the nature of the anti-tumor response required for clinical benefit. Until recently, dissecting the diversity and quantity of TCR clonotypes within the tumor microenvironment has been difficult because of wide variations in tumor antigen-associated (TAA) expression, human leukocyte antigen (HLA) haplotypes, and the individually different spontaneous immune responses against TAAs [14]. High-throughput next-generation sequencing has made it possible to read the entire CDR3 to uniquely identify specific T cell clones and to estimate the absolute frequency of T cell clones in tumor tissue from the copy number of TCR sequences [13]. Recent studies have analyzed the TCR repertoire in the context of autoimmune disorders [15, 16], infection [17], immunodeficiency [18], and cancer [19, 20]. These studies have compared TCR repertoires in different tissues (e. g. peripheral blood versus diseased sites), and across time points. In addition, immunotherapy trials in melanoma and pancreatic cancer patients have demonstrated the potential for TCR repertoire profiling to serve as a biomarker of clinical response and toxicity [21, 22].

The importance of TCR repertoire in shaping anti-tumor immunity in ovarian cancer was recently demonstrated using unbiased functional analysis of TCR repertoires from TILs derived from two patients [23]. Tumor reactivity was revealed in 0–5% of tested TCRs indicating that the vast majority of T cells infiltrating ovarian tumors were irrelevant for tumor recognition. To determine how the TCR repertoire of TILs shapes the prognosis of ovarian cancer patients, we utilized a new computational method [24] for *de novo* assembly of sequences from CDR3 regions using paired-end RNA-seq data from the Cancer Genome Atlas (TCGA) study of high-grade serous ovarian cancers [25]. We then applied deep TCR-sequencing to a large number of paired tumor and peripheral blood mononuclear cell (PBMC) specimens from patients with ovarian cancer. We examined TCR repertoire in the context of (i) the degree of tumor infiltration by T cells, (ii) spontaneous immune responses against *bona fide* TAAs, and (iii) clinical outcome. Importantly, we also found that the degree of divergence of the peripheral from the TIL repertoire, and the presence of detectable spontaneous anti-tumor immune responses are important determinants of clinical outcome. These immunological phenotypes defined by the TCR repertoire may provide useful insights for identifying “TIL-low” ovarian cancer patients that may respond to immunotherapy.

## RESULTS

### Patient characteristics

As described in the initial report [25], TCGA ovarian patients were all high-grade serous ovarian cancer treated with surgery and adjuvant platinum based chemotherapy. More than 86% were high stage, the median age was 59.8 years, and 72.1% were optimally debulked (< 1 cm) with 22.7% achieving full resection (no macroscopic disease). The median progression-free survival (PFS) was 17.5 months (15.4–18.5), and the median overall survival (OS) was 44.1 months (39.6–47.7, 95% CI), with a 5-year survival rate of 31.3% (26.5–37.0%).

The characteristics of the RPCI cohort are shown in Table 1. Whereas TCGA cohort was restricted to high-grade serous histotype, patients with non-serous tumors were included in the RPCI cohort because of availability of paired PBMC and tumor specimens. Patients in this cohort were also typical of advanced ovarian cancer cases: the median age of diagnosis was age 62 (range 20 to 88), most patients were high stage (IIIC, IV; 77%) with serous histology (66%). All women underwent maximal debulking surgery (78% were optimally debulked, with 36% complete resection) followed by platinum-based chemotherapy. The median duration of follow-up for all the patients was 33.5 months (91/99 patients have observed deaths or at least 36 months of follow-up). While the majority (58%) of patients were disease-free after primary therapy, 34% recurred within a median 8.6 months of their initial surgery. Median progression-free survival was 15.4 months and median overall survival was 48.7 months.

**Table 1:** RPCI cohort patient characteristics stratified by stage and infiltration

Total *N* = 99	A. Low Stage *N* = 22	B. High Stage High Infiltration *N* = 38	C. High Stage Low Infiltration *N* = 39	*p*-value (A vs. B, C)	*p*-value (B vs C)
TIL infiltration (median)					
CD3 (lym/mm^^^2)	106.7	399.3	39.0	0.60	^**^
CD8 (lym/mm^^^2)	20.7	105.3	3.1	0.50	^**^
CD8/CD3	38.5%	24.2%	32.3%	0.20	0.10
Age					
Median	57.5	60.5	66.0	0.40	0.02
Grade %3+	61.9%	92.1%	71.8%	0.10	0.04
Histology % Serous	45.5%	84.2%	61.5%	0.03	0.05
Debulking Status					
% No residual disease	86.4%	23.7%	21.1%	< 0.001	0.99
% Optimal	100.0%	76.3%	71.1%	0.02	0.80
Response after Treatment					
% Complete	95.5%	64.9%	36.1%	< 0.001	0.03
Platinum status					
% Sensitive	100.0%	66.7%	28.1%	0.002	0.004
Survival Times					
Progression-Free survival	^##^	15.4	9.7	< 0.001	0.055
Overall survival	^##^	52.9	25.0	0.002	0.013

^**^Stratifying factor. ^##^No median Kaplan-Meier estimate. TIL: Tumor infiltrating lymphocytes.

### Pretreatment TIL infiltration and tumor antigen burden is associated with survival

We first sought to establish in TCGA and RPCI cohorts whether the degree of T-cell infiltration was a favorable prognostic marker as previously reported [5, 26]. For TCGA cohort, we selected CD3^+^-based markers for this analysis as previously described [27]. These markers included mRNA expression of *CD3*, *CD8*, *GZMB*, and *IL2*. In addition, we focused the analysis of CD3^+^-based markers in the context of tumor expression of shared antigens belonging to the cancer-testis (CT) antigen family, because their expression is normally restricted to germ cells but they become aberrantly expressed in several tumors types, including ovarian cancer [28]. We included the average expression of 91 unselected CT antigens to form an antigen burden score [29]. While the expression of CD3^+^ markers was associated with a modest increase in PFS (19.0 vs 22.8 months, *p* = 0.0376) and a large increase in OS (47.6 vs. 70.6, *p* = 0.0008), stratification by above median antigen burden magnified the effect among infiltrated patients (PFS 21.1 vs 29.4 months, *p* = 0.0373, OS 60.4 vs. 84.8 months, *p* = 0.0038) (Supplementary Figure 1A–1C). The observed benefit was specific to patients with both heavy CT antigen burden and significant TIL infiltration (PFS 29.4 vs. 19.5, *p* = 0.0054; OS 84.8 vs 51.9 months, *p* = 0.0034). The volcano plot in Supplementary Figure 1D shows upregulated genes in this category to include CXCL9/10, IFI6/35 and ISG15 (interferon induced). These results indicate that the expression of CT antigens, when accompanied by high TILs, produces an immunogenic tumor microenvironment that is associated with improved PFS and OS in ovarian cancer patients. Interestingly, we observed that tumors expressing CTAGE5 and LAGE3 had the highest T-cell infiltration (Supplementary Figure 1E). LAGE3 belongs to the ESO/LAGE gene family, members of which are clustered together on chromosome Xq28, and have similar exon-intron structures [30]. However, unlike the other family members which are normally expressed only in testis, LAGE3 is also ubiquitously expressed in somatic tissues. Although NY-ESO-1 expression is associated with intermediate levels of T-cell infiltration in TCGA cohort (Supplementary Figure 1E), we focused on this antigen for additional studies in the independent RPCI cohort because of its unique characteristics of tissue restricted expression and inherent immunogenicity. Moreover, NY-ESO-1 is the prototypic CT antigen in several cancer vaccine [31–35] and adoptive T-cell therapy studies [36, 37].

In the RPCI cohort, we found that the degree of T-cell infiltration measured by immunohistochemistry (IHC) was an important prognostic marker, in accordance with previous reports [5, 26] and TCGA cohort. The densities of tumor-infiltrating CD8 and CD3 cells were strongly correlated (rho = 0.85, *p* < 0.001) and both were correlated with prognosis. CD3 infiltration was associated with favorable PFS (log scale, stage-adjusted HR = 0.59, 95% CI: 0.43–0.83) and OS (0.44, 0.28–0.69) (Table 2). Further, high levels of infiltration were associated with platinum sensitivity (CD3 ratio of sensitive to resistant 1.25×, *t*-test *p* = 0.0011; CD8, 1.29×, *p* = 0.0051) (Table 1). There was no significant difference in the degree of T cell infiltration between serous and non-serous tumors.

**Table 2:** Prognostic features of the ovarian cancer TCR repertoire

	Hazard Ratio (95% CI, Cox Model)	
	PFS	OS
Infiltration^*^		
CD3 (log lym/mm^2^)	0.59 (0.43–0.83)	0.49 (0.33–0.72)
CD8 (log lym/mm^2^)	0.57 (0.37–0.86)	0.46 (0.28–0.77)
Focus: Diversity/TIL^*^		
#Clones/CD3	5.32 (1.98–14.30)	5.46 (2.03–14.66)
#Clones/CD8	4.14 (0.53–32.37)^N/S^	17.49 (1.59–^***^)
Overlap^**^		
Clones	1.35 (1.06–1.72)	1.41 (1.10–1.80)
Reads	1.04 (0.78–1.38)^N/S^	1.27 (0.94–1.71)^N/S^
Diversity Ratio^**^		
PBMC/TIL	0.73 (0.55–0.97)	1.01 (0.74–1.39)^N/S^

All factors scaled to unit standard deviation. ^*^Stage adjusted, ^**^CD3 adjusted, ^***^Upper bound omitted due to size, ^N/S^Not significant (*p* > 0.05).

As stage is known to be the strongest predictor of survival in ovarian cancer, we stratified patients into low stage (Stages I, II, IIIA, IIIB) and high stage (IIIC, IV) cases. We further separated the high stage cases by degree of CD3 infiltration to further characterize the high and low-infiltrating cases. Highly infiltrated, high-stage disease were more commonly high grade and serous and these patients were an average of 6 years younger (Table 1). Clinically, high-stage patients had the same rate of optimal debulking; however, highly-infiltrated patients are more likely to demonstrate complete response to frontline chemotherapy, and were more likely to be platinum sensitive after follow-up. Median PFS and OS increased by 5.7 and 27.9 months in high stage-highly infiltrated patients, compared with high-stage-low infiltrated patients (Table 1). These results are consistent with our previous report and other studies [5, 38] that the degree of T-cell infiltration prior to treatment of ovarian cancer patients are strongly associated with response to frontline therapy, PFS and OS even after accounting for stage.

### Composition of β-CDR3 repertoire is associated with prognosis

While the average degree of TIL infiltration was associated with prognosis, there remained considerable variation in outcome. Previous studies have shown that TILs represent a heterogeneous population consisting of tumor-specific and bystander T cells [8]. We hypothesized that the specific composition of tumor-targeting clonotypes in the TIL population and periphery might influence the prognostic effect and provide indication of immunologically-driven tumor response. Starting with TCGA cohort, we investigated CDR3 assemblies as recently described by Li *et al.* [24]. We identified 39,272,923 TCR-mapping RNA-seq reads comprising 25,215 unique amino acid sequences in 416 ovarian cancers. Upon manual inspection, we noted that many of the reads were fragments containing common V-gene sequences only or J-gene sequences only. Therefore, we restricted amino acid sequences to those that are likely to contain a complete CDR3, encoding the initial cysteine and the terminal phenylalanine. Using this filter, we observed 13,870,967 TCR mapping RNA-seq reads comprising 9,990 amino acid sequences in 394 ovarian cancers from TCGA study. The distribution of filtered clone lengths was representative of peripheral blood CDR3 regions [39]. We observed one clone common to 20 repertoires (5.1%, 20/394) suggesting that ovarian cancer repertoires are mainly private. This common clone was not associated with prognosis.

Summary features of the TIL repertoire were strongly associated with prognosis in TCGA cohort (Supplementary Table 1). While (Shannon’s) entropy and the number of unique clones (diversity) were associated with reduced survival hazard (HR = 0.76, *p* < 0.01), clonality (1-Pielou’s index) was not (HR = 1.07, *p* = 0.462) suggesting that the range of clonotypes and not their usage was important. We defined the strength of monoclonality as the relative frequency of the most common clone (TOP1) and observed that the strength of monoclonality (TOP1) was associated with increased hazard (HR for OS = 1.36, *p* < 0.01). As expected, the degree of infiltration (measured by the number of mRNA TCR reads) was associated with reduced hazard (HR = 0.77, *p* < 0.01).

### Validation by deep sequencing shows high TIL diversity is associated with high stage, high infiltration disease

To validate the findings from TCGA cohort and understand the complex effect of clone diversity on prognosis, we performed deep TCR-β chain sequencing on total 198 samples of matched TIL and PBMC from 99 patients in the RPCI ovarian cancer cohort (Supplementary Table 2). Each patient’s TIL TCR repertoire contained between 224 and 94,770 (Q1: 2,918; median: 11,590; Q3 15,190) unique clones (Supplementary Table 3). Between 65.3% (Q1) and 84.0% (Q3) of reads were captured by the most common 1,000 clones implying that most samples have a significant fraction of low-frequency reads. The number of reads in the tumor specimens (17,978-13,368,648) was associated with the density of CD3^+^ T-cell infiltration (Spearman’s rho = 0.340, *p* = 0.001), indicating concordance between IHC-measured infiltration and depth of TCR sequencing. Consistent with TCGA cohort, the RPCI cohort’s TIL and PBMC repertoires showed the same trend of prognostic effects (Entropy, and Diversity associated with reduced hazard; TOP1 with increased hazard; clonality no effect), however they did not reach statistical significance (Supplementary Table 1).

We investigated these associations further by stratifying based on CD3 and CD8 infiltration measured by IHC. The relative frequencies of clones were not different across patient categories (low stage, high stage, high infiltration; and high stage, low infiltration) (Supplementary Figure 2). There was no difference in clonality across histologies and stage/infiltration strata suggesting the overall composition of the repertoire is not different (Table 3). Compared with peripheral blood, clonality doubles in the TIL compartment of all three categories of ovarian cancer patients (TIL 0.22 vs. PBMC 0.12, paired *t*-test *p* < 0.001), indicating a more specific or focused response at the tumor site. In the TIL repertoire, the number of unique clones doubled for high stage disease (5,770 vs. 13,280, *t*-test *p* = 0.003) while the number of clones in the PBMC repertoire was not different across high/low stages (85,206 vs. 83,411, *p* = 0.845). Despite this tumor-site doubling, clonality was not associated with PFS (Stage adjusted, Cox model, *p* = 0.93) or OS (*p* = 0.18) confirming that the pattern of usage of specific clones did not drive prognosis.

**Table 3:** T cell receptor properties stratified by stage and infiltration

	A. Low Stage *N* = 22	B. High Stage High Infiltration *N* = 38	C. High Stage Low Infiltration *N* = 39	*p*-value (A vs. B, C)	*p*-value (B vs C)
Clonality (1-Norm. Entropy)					
TIL	0.189	0.204	0.210	0.827	0.455
PBMC	0.095	0.078	0.087	0.875	0.234
Diversity (Median #Clones)					
TIL	2,828	11,549	7,317	0.003	0.136
PBMC	86,190	94,513	65,561	0.845	0.019
Depth (Median #Reads)					
TIL	511,068	2,791,190	1,864,132	0.069	0.132
PBMC	3,651,511	3,877,524	3,468,589	0.582	0.267
Focus: Diversity/TIL					
#Clones/CD3	1.80	1.53	2.80	0.929	< 0.001
#Clones/CD8	3.28	1.95	22.72	0.645	< 0.001
#Clones/CD4	7.80	6.83	7.49	0.330	0.048

### Specific public clones are not associated with prognosis

**Figure 1:**
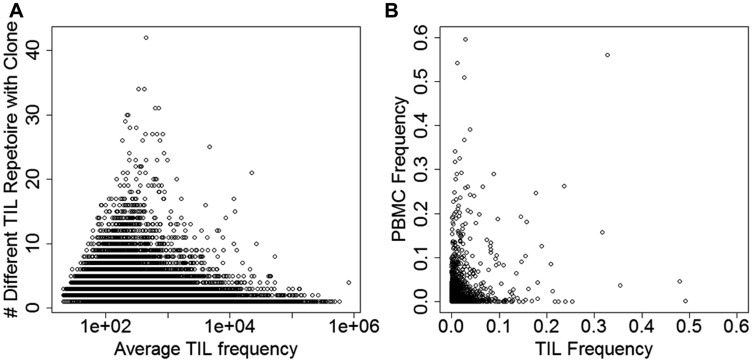
Deep TCR sequencing clones shared across people and tissues in the RPCI cohort. (**A**) Degree of sharing and average frequency of tumor-infiltrating lymphocyte (TIL) clones across all patients. (**B**) Frequency of specific clones in matched peripheral blood (PBMC) and TIL repertoires combined over all patient samples.

Considering that summaries averaging tens of thousands of clones might obfuscate signal, we investigated high-frequency clones and clones common to multiple TIL repertoires (i.e., across patients). Out of 670,723 unique TIL clones, 7 clones were found in at least 30 repertoires and 18 were found in at least 25 repertoires (Figure 1A). We reasoned that a subset of peripheral and TIL TCR repertoire would overlap, and the degree of overlap may reflect clonal expansion of tumor-reactive T cells. We observed that between the TIL and PBMC repertoires, highly frequent TIL clones were often rare in the PBMC repertoire and vice versa (Figure 1B).

The association between the top 18 common clones and PFS suggested one prognostic association: the frequency of clone CASSLTDTQYF was associated with increased PFS hazard (standardized to unit standard deviation; stage-adjusted HR = 1.53, 95% CI: 1.16–2.03, Bonferroni *p* = 0.046) and OS hazard (HR = 1.56, 1.18–2.06, Bonferroni *p* = 0.032). This effect appeared to be specific to one particular TIL repertoire, although we could not rule out statistical artifacts after data transformation (Supplementary Table 4). In summary, no single, public clone was obviously associated with prognosis.

### Focused clonal repertoire of TIL infiltration is associated with good prognosis in the validation cohort of ovarian cancer patients

**Figure 2:**
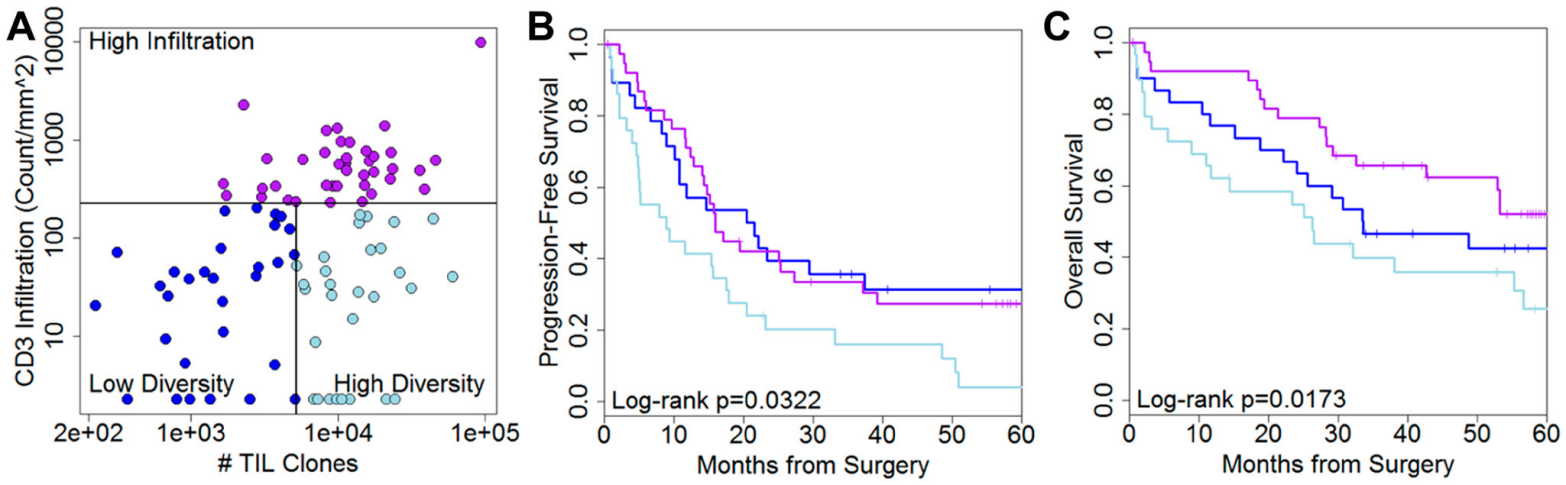
CD3 infiltration by clone diversity. (**A**) Scatterplots and Kaplan–Meier plots for (**B**) progression-free survival (PFS) and (**C**) overall survival (OS) show decreased hazard of progression and mortality due to weak infiltration by a focused (low diversity) repertoire. Thresholds are selected for three evenly sized groups.

In addition to specific clone level response, we sought to further clarify the relationship between IHC-measured TIL infiltration and TCR repertoire. Given that we had noted high TIL frequency as a strong prognostic factor in both TCGA and RPCI cohorts, we stratified RPCI patients with weakly infiltrated tumors by the diversity of their repertoire (Figure 2A) into three evenly sized groups. Surprisingly, patients with weakly-infiltrated tumors but low diversity repertoires (that is, focused repertoires) had PFS (Figure 2B) indistinguishable from highly-infiltrated tumors (median 21.0 months versus 15.9 months, log-rank *p* = 0.945). Both of these groups had nearly double median survival (16.5 versus 8.8 months, *p* = 0.004; 3 group log-rank *p* = 0.0322). Overall survival times (Figure 2C) fit this pattern with low diversity repertoires (26.3 months) versus high diversity and high infiltration (53.2 months, three group log-rank *p* = 0.0173).

**Figure 3:**
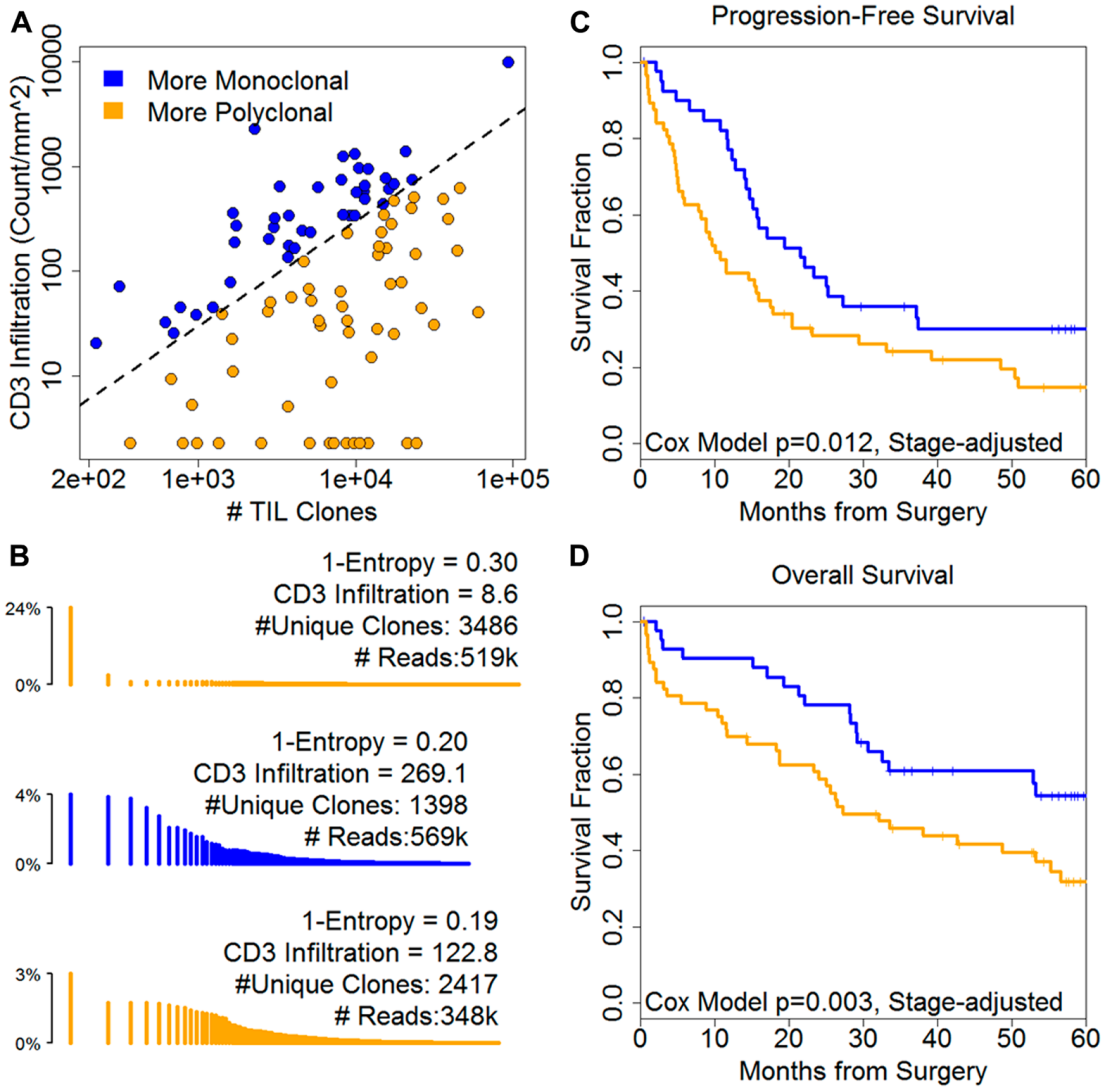
Infiltration and repertoire diversity combine to describe prognosis. (**A**) The ratio of CD3 infiltration to TIL clone diversity defines monoclonal versus polyclonal individual repertoire distributions. (**B**) Representative histograms in which each line reflects a unique TCR clone; clones are ordered by descending frequency on a log scale. Color summarizes both degree of infiltration and repertoire diversity. Low levels of infiltration relative to clone diversity are associated with poor prognosis by (**C**) PFS and (**D**) OS. 1-Entropy is a measure of clonality; clones are unique amino acid sequences; reads are counts of a given clone.

To simplify the effect and consider sensitivity to the selected thresholds, we examined the ratio of the number of unique TIL clone versus CD3 TILs. Ratios closer to zero indicate more focal, monoclonal repertoire and large ratios imply a polyclonal repertoire (Figure 3A). Representative monoclonal and polyclonal repertoires (Figure 3B) illustrate that an array of methods are required to balance infiltration, composition of that infiltration (clonality or 1-entropy), the diversity and the read depth (Supplementary Figure 3). More polyclonal clone/CD3 TIL ratios were associated with shorter PFS (5.3, 2.0–14.3, Figure 3C) and OS (HR = 5.5, 95% CI: 2.0–14.7, Figure 3D). Polyclonal clone/CD8 ratios were associated with OS only (HR = 17.5, 1.6–192.0) (Table 2). More polyclonal ratios were associated with platinum resistance for both TIL markers (CD3, *t*-test *p* = 0.001; CD8, *p* = 0.006). These results indicate that TCR diversity is a determinant of the prognostic influence of TILs in ovarian cancer. In addition, integrating IHC measurements with TCR sequencing data further improves the prognostic significance of the TIL repertoire in ovarian cancer.

### Polyclonal peripheral repertoire is a poor prognostic factor

We observed that unfocused TIL repertoires were matched by a similarly unfocused repertoire in the periphery (a highly diverse PBMC relative to read depth), suggesting that while the peripheral repertoire might possess clonotypes found within the tumor, these clones may fail to preferentially accumulate at the tumor site. To test his hypothesis, we considered (i) the overlap between each individual’s TIL and PBMC compartments and (ii) the relative diversity of TIL versus PBMC clones.

**Figure 4:**
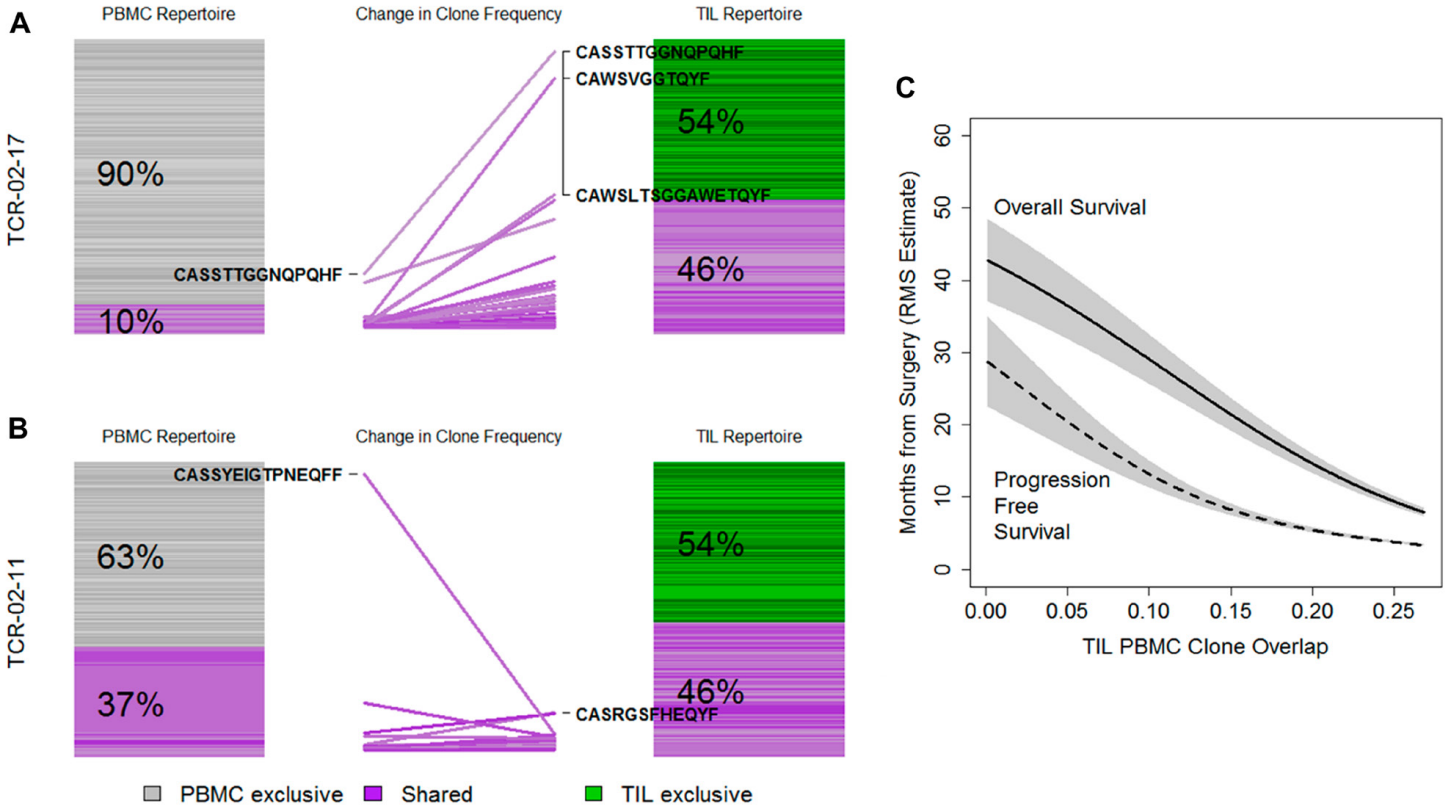
Repertoire changes at the tumor site and in the periphery. (**A**) PBMC (left) and TIL repertoires (right) for one patient with change in individual clone frequency (center). Bars are individual clones and their widths reflect the number of reads attributed to each clone. Amino acid sequences for high-frequency clones are shown. (**B**) As above, this patient has a similar read overlap but a dramatic shift in clone usage. (**C**) Restricted mean survival (RMS) overall survival and PFS and OS estimates for the effect of clone-level overlap between TIL and PBMC repertoires.

We illustrate the concept of PBMC and TIL overlap as well as exclusive clones for the patient with the highest amplification of overlapping clones (Figure 4A) and lowest amplification (Figure 4B) where each bar is a clone and its width is proportional to the clone’s frequency in the repertoire. Grey clones are PBMC exclusive, green are TIL exclusive and purple clones are found in both repertoires. Clones with the largest individual frequency change between repertoires are highlighted. Across patients, we found few clones common to multiple repertoires (Supplementary Figure 4) and, again, clones tended to be either shared or highly frequent but not both.

Within a given patient, repertoire overlap can be based on unique clones (assessing the chance that a randomly selected PBMC clone is also found in the tumor repertoire) or the number of reads (assessing the fraction of the PBMC repertoire that is known to infiltrate the tumor, given multiple copies of a particular TIL clone). Clone level overlap averaged 2.27% (range: 0.10–19.40%) while the read level overlap averaged 20.3%, (range: 1.01–70.0%) suggesting that tumor-infiltrating clones may make up a disproportionate, but variable, amount of the peripheral repertoire. We noted that the fraction of shared clones is higher in the TIL compartment than the periphery (Supplementary Figure 5) consistent with the idea that the TIL compartment is more selective or exclusive than the peripheral repertoire.

Clone overlap between TIL and PBMC repertoires was strongly associated with CD3 infiltration (rho = 0.340, *p* = 0.001) but not CD8 infiltration (rho = 0.145, *p* = 0.2). The degree of overlap was strongly associated with shorter PFS (HR = 1.35, 1.06–1.72, *p* = 0.016) and OS (1.41, 1.10–1.80, *p* = 0.006) after accounting for CD3 infiltration (Figure 4C). Read-level overlap was not associated with OS (*p* = 0.12), PFS (*p* = 0.80), CD3 (*p* = 0.51) or CD8 (*p* = 0.43) infiltration. Together, these results implied that infiltration by a restricted TIL repertoire is a positive prognostic sign, given a specific level of infiltration.

To account for individual differences in repertoire size, we considered the relative diversity of TIL and PBMC repertoires using the ratio of PBMC clones to TIL clones adjusting for read depth. This ratio was not associated with CD3 infiltration (*p* = 0.46), so it forms a prognostic dimension independent of infiltration. Accounting for infiltration and stage, the ratio was associated with PFS (HR = 0.73, 0.55–0.97, *p* = 0.031) but not OS (1.01, 0.74–1.39, *p* = 0.950). The direction of effect implies that comparatively diverse TIL repertoires (polyclonal) continue to be markers of poor prognosis.

### Prognostic effect of peripheral repertoire depends on humoral response to NY-ESO-1

The restricted repertoire infiltrating the tumor leads us to hypothesize that an antigen-specific or antigen-shaped repertoire might drive clonal restriction and ultimately prognosis. Revisiting our TCGA analysis of CT antigens, LAGE3 showed the highest expression and had significantly higher expression in CD3^+^ cases (*t*-test *p* < 0.001, Supplementary Figure 1E). LAGE3 is a close analog of NY-ESO-1, probably the most immunogenic tumor antigen to date, and has been extensively explored as a target of immune recognition in several clinical trials in ovarian cancer [28, 33].

**Figure 5:**
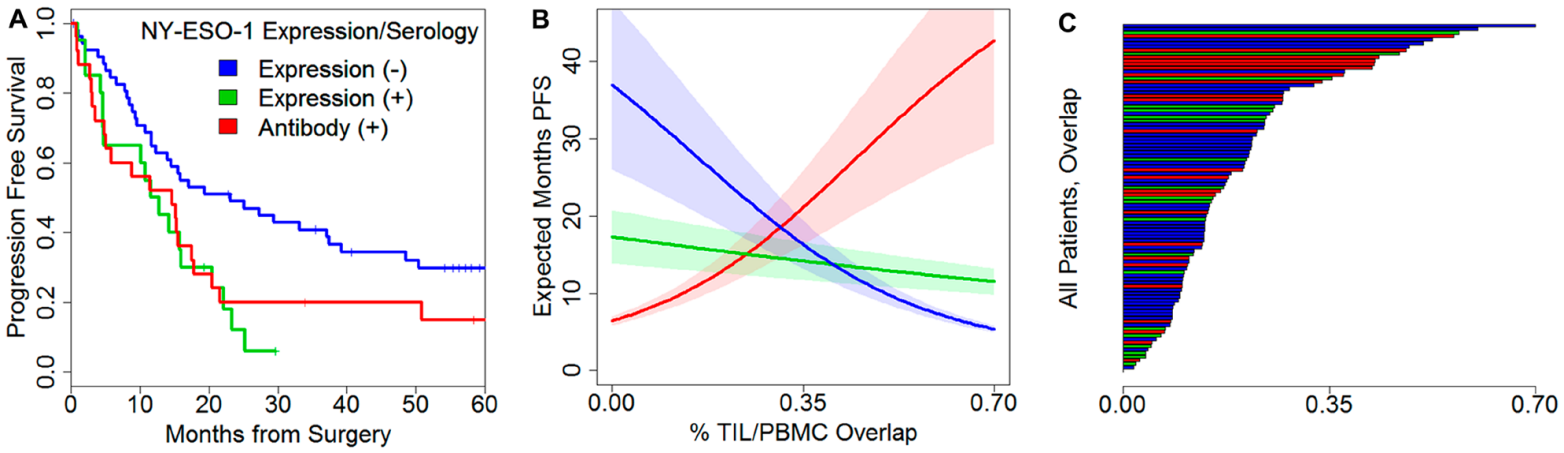
Prognostic effects of NY-ESO-1 expression and serology with TCR repertoire features. (**A**) Kaplan–Meier plot evaluating PFS based on NY-ESO-1 expression and NY-ESO-1 serology, unadjusted for TCR features; *p* = 0.013. (**B**) Expected restricted mean PFS as a function of TIL/PBMC overlap. (**C**) Sorted bar chart of overlap across patients colored by expression/serology.

Because humoral response to NY-ESO-1 is tightly associated with specific CD8^+^ and CD4^+^ T-cell responses against the antigen [40, 41] and is a frequently observed antigen in ovarian cancers [28, 42], we evaluated NY-ESO-1 serology as a surrogate for the presence of T cell responses. NY-ESO-1 serum antibodies were detectable in 25/99 (25%) of the patients, and 38/99 patients (38%) showed spontaneous antibody responses against at least one of NY-ESO-1, MAGE-A1, MAGE-A3, and p53 antigens. NY-ESO-1 seropositive patients were significantly older (68 versus 60 years, *p* = 0.007) and had higher pathological grade disease (95.8% versus 71.6%, *p* = 0.029, Supplementary Table 3). Without considering the TCR repertoire, patients with NY-ESO-1 negative tumors had significantly longer PFS (23 versus 12 versus 14 months, *p* = 0.013, Figure 5A).

While the diversity of clones did not differ between seropositive and seronegative patients (PBMC *t*-test *p* = 0.11, TIL *p* = 0.67), the number of PBMC reads decreased by 23% in seropositive patients (mean 4.28 million reads versus 3.46 million reads, *t*-test *p* = 0.009). This suggested that antigen responses might be apparent by considering clone usage and not diversity. We reasoned that the PBMC exclusive fraction, i.e. the subset of the peripheral repertoire spent on clones that do not traffic to the tumor, may capture the prognostic effect of antigen-specific response. Adjusting for stage and depth of infiltration, Cox model regression did find that seropositive patients with greater levels of PBMC exclusive clones had poorer prognosis (PFS, interaction deviance test *p* = 0.011, OS *p* = 0.022, Supplementary Table 5). We were surprised to see that greater exclusivity was associated with better prognosis in seronegative patients (Supplementary Figure 6A, 6B). To further delineate the impact of concomitant presence of tumor reactive clones in the tumor and periphery, we evaluated the degree of TIL/PBMC overlap. We found that these sharply contrasting antigen-stratified effects were again present (Supplementary Figure 6C, 6D); in particular, expression negative patients prognosis increased with less overlap while seropositive cases improved with greater overlap (Figures 5B, Supplementary Figure 6C, 6D). The set of expression positive, seronegative patients had a neutral prognostic effect (Figure 5B). The degree of TIL/PBMC overlap for all individual patients is shown in Figure 5C. Together, these results indicate that the impact of TIL/PBMC clonal overlap is shaped by antigen status and tumor immunogenicity as measured by the presence of spontaneous immune response to tumor antigens.

## DISCUSSION

There is compelling evidence that features of the immunological response to tumor such as the density of TILs, ratios of T-cell subpopulations, expression of immune checkpoint regulators, and cytokines [1, 5, 38, 43–47], are useful prognostic features for ovarian cancers; and a comprehensive immunological scoring system is under development [48]. Among these parameters, the quantity and quality of T cells within the tumor is one of the most crucial for effective tumor destruction. Despite the strong correlation between high T-cell infiltration (“TIL-high”) and improved overall clinical prognosis in ovarian cancer [5, 26, 49], we observed that a proportion of patients with “TIL-low” tumors exhibit clinical outcomes that are similar to “TIL-high” patients. This raises the possibility that the T cells of these “TIL-low” patients with favorable prognosis have unique attributes. To address this, we focused on the degree of T-cell clonality in TIL and PBMC, clonal overlap of TIL/PBMC, and spontaneous immune responses to NY-ESO-1 and other shared tumor antigens (MAGE-A1, MAGE-A3, MAGE-A4 and p53). We utilized a novel method for *de novo* assembly of sequences from CDR3 regions using paired-end RNA-seq data from TCGA ovarian cancer cohort, and validated our findings in a large independent cohort of ovarian tumors and their matching PBMC from 99 ovarian cancer patients. The key findings of our study are that (i) tumor infiltration by T cells when accompanied by high CT antigen burden is associated with improved PFS and OS; (ii) the number of TCR sequenced reads is directly related to TIL infiltration; (iii) more diverse infiltration, measured by the number of unique TCR sequences for a fixed level of infiltration, is a prognostic biomarker of poor overall survival; while infiltration by a restricted TIL repertoire is a positive prognostic sign, even in patients with “TIL-low” tumors; (iv) an increasing degree of overlap between the TIL and peripheral repertoire is a further prognostic biomarker of poor outcome; and (v) the presence of detectable tumor antigen-specific immune response further shapes the prognostic effect of the peripheral repertoire, such that patients with pre-existing immune responses against TAAs (e. g. NY-ESO-1) benefit from increasingly clonal infiltration. Collectively, these results indicate that the prognostic significance of TILs is shaped by the specific clones infiltrating tumor and not only their numbers.

In this study, we initially confirmed that TILs play an important role in anti-tumor responses as evident from the correlation between prognosis and the number of tumor-infiltrating CD3^+^ and CD8^+^ cells. However, the impact of T-cell diversity in shaping the prognostic significance of TILs was striking. In this regard, we observed that even in patients with weak infiltration by T cells but with focused repertoires, the clinical outcomes (PFS and OS) were similar to patients with highly infiltrated tumors. We translated these findings into the ratio of the number of unique TIL clone versus CD3 TILs and found that clone/CD3 TIL ratio may represent a novel prognostic biomarker in ovarian cancer.

Surprisingly, we found that higher TIL clonality confers favorable prognosis in patients with detectable spontaneous immune responses against TAAs and it is a poor prognostic factor in patients without detectable immune responses to the TAAs. Considering that humoral immune response to NY-ESO-1 is tightly associated with specific CD8^+^ and CD4^+^ T cell responses against the antigen [41, 50], our results suggest that high-frequencies of dominant TAA-specific T-cell clones present at the time of diagnosis are clinically relevant for mediating improved survival of ovarian cancer patients. It is likely that when patients develop anti-tumor immune responses, TAA-restricted oligoclonal infiltration provides strong anti-tumor effects. In contrast, for seronegative patients, a more diverse or polyclonal T-cell repertoire is associated with favorable prognosis. A possible explanation is that polyclonal TCR repertoires may represent subdominant tumor-reactive clones or non-tumor-reactive population of T cells. Interestingly, we observed that patients with unfocused TIL repertoires were more likely to have unfocused repertoire in the periphery, and that the degree of overlap between peripheral T-cell and TIL repertoire is strongly associated with prognosis. These results suggest that while the peripheral repertoire might possess clonotypes found within the tumor, these clones may fail to preferentially accumulate at the tumor site. However, the effect reverses between NY-ESO-1 seropositive and seronegative groups: more overlapping TCR clones in the periphery are associated with favorable prognosis in seropositives and poor prognosis in seronegatives. It is likely that the presence of tumor-associated T-cell clones in the periphery provides strong anti-tumor immunity that control systemic tumor metastasis. The opposite effect in seronegative patients may indicate that the TIL repertoire in seronegative patients may be irrelevant for tumor control. Thus, the correlation between TIL frequency, clonality and clinical outcome is further shaped by the presence of spontaneous anti-tumor immune responses to *bona fide* tumor antigens such as NY-ESO-1.

There are a number of limitations to our study. First, although we analyzed immune responses against a pre-selected set of *bona fide* TAAs (NY-ESO-1, MAGE-A1, MAGE-A3, and p53), these do not represent the full range of tumor antigens expressed in ovarian cancer. Seronegative patients may in fact have immune responses against other TAAs and neoantigens. Indeed, recent studies [51, 52] suggest that the mutational neoantigen landscape present in melanomas is associated with clinical benefit from checkpoint inhibitors. This limitation is somewhat mitigated by the flexibility of sequencing technology: the general TCR analysis that we have conducted is not biased for our selection of antigens. Second, although we show that clone/CD3 TIL ratios may represent novel prognostic biomarkers in ovarian cancer, subset analysis of TILs (Th1, Th2, Th17, Tregs) may provide additional insight into the clonal lineage of effector or regulatory cells that drive prognosis. Third, because of sample availability, the RPCI cohort included serous and non-serous tumors, whereas TCGA cohort was restricted to serous cases. Although we found that the degree of T cell infiltration and clonality were not statistically different between serous and non-serous tumors, it is important to investigate whether antitumor role of TILs is different between histotypes. Finally, the study population was not treated with any form of immunotherapy, so it remains unknown whether these prognostic markers will be able to select patients who are likely to respond to immunotherapy.

Despite these limitations, this study highlights the extraordinary diversity of the T-cell repertoire in ovarian cancer patients, and demonstrates that pre-existing immunity against cancer antigen is a critical prerequisite to correctly understand the prognostic significance of the T-cell repertoire in the tumor and peripheral blood of patients with ovarian cancer. We have distilled TCR repertoire information into candidate biomarkers that may critically influence the prognosis of ovarian cancer patients. Conceptually, ovarian cancers may not fit into the classic paradigdm of ‘cold’ and ‘hot’ based on the number of T cells they contain, but also by the TCR repertoire information, which serves as a surrogate for tumor recognition. The latest technologies put these prognostic features in clinical reach not only for predicting prognosis but potentially for determining the best immunotherapeutic strategy for each patient.

## MATERIALS AND METHODS

### Patients and sample preparation

Medical records, tumor, and blood specimens were collected from 99 patients with a primary diagnosis of ovarian, fallopian tube, or peritoneal cancer treated at Roswell Park Cancer Institute (RPCI) between 2001 and 2012 under Institutional Review Board (IRB) protocol CIC 02-15 (RPCI cohort). Tumor specimens from this RPCI cohort were collected at the time of primary debulking surgery, frozen in liquid nitrogen and stored at -80°C. PBMCs and serum were obtained from peripheral blood and stored at -80°C. Patients included in this study were those with adequate biospecimens (tumor, PBMCs and serum) for analysis. DNA was extracted from the frozen tissue and PBMCs using the GenFIND DNA extraction kit (Agencourt, Pasadena, CA) per the manufacturer’s instructions. All pathology specimens were reviewed at RPCI by experienced gynecologic pathologists and tumors were classified according to the WHO criteria [53]. Prior to surgery no patients received neo-adjuvant chemotherapy and subsequent to surgery all patients received adjuvant platinum/taxane-based chemotherapy.

### Tissue microarray, immunohistochemical staining and measurement of serum antibodies

Tissue microarrays (TMA) were constructed using formalin-fixed and paraffin-embedded tumor tissues, stained for CD3 and CD8, and scored as described in our previous studies [5, 54]. CD4 levels were imputed as the difference between CD3 and CD8 level. Serum antibody levels against NY-ESO-1, MAGE-A1, MAGE-A3, and p53 were measured by ELISA using E. coli-derived recombinant proteins as described previously [55]. Reciprocal titers greater than 100 were considered significant.

### TCR analysis of ovarian carcinoma samples from The Cancer Genome Atlas (TCGA)

We downloaded BAM formatted (hg19 aligned) RNA-seq data for 369 TCGA ovarian carcinoma patients achieving complete response after adjuvant therapy from the Cancer Genomics Hub. We used TRUST (Tcr Receptor Utilities for Solid Tissue) to infer the complementarity-determining region 3 (CDR3) sequences of tumor-infiltrating T cells in these TCGA ovarian carcinoma samples. TRUST performed de novo assembly on the hypervariable CDR3 and reported contigs containing the CDR3 DNA and amino acid sequences, followed by realigning the contigs to IMGT reference gene sequences to report the corresponding variable (V) or joining (J) genes [24]. Based on the TRUST output, we calculated clonotypes per kilo reads (CPK, the number of unique CDR3 calls in each sample normalized by the total read count in the TCR region) as a measure of clonotype diversity of T cell repertoire in each sample. *URLs* TCGA data portal, https://tcga-data.nci.nih.gov/tcga/tcgaDownload.jsp.

### TCR sequencing and coverage statistics

TCR-β chain CDR3 regions of patients in the RPCI cohort were sequenced and analyzed using the ImmunoSEQ platform (Adaptive Biotechnology, Seattle, WA) as previously described [13, 56]. Observed reads were normalized to account for different PCR efficiency across different primer sets and low frequency reads (fewer than 20 copies) were omitted as low-level noise (Supplementary Table 3). The copy number was defined as a normalized count of reads per each unique CDR3 amino acid sequence.

### Statistical methods

Cox’s proportional hazards model was used for time-to-event regression and the effects are described as hazard ratios with 95% confidence intervals where the variable of interest is scaled to unit standard deviation, unless otherwise noted, to make variables on different scales comparable. The restricted mean survival (RMS) plots show the parametric effect of these models as a function of covariate levels with survival capped at 60 months to emphasize the clinically important time period for ovarian cancer [57]. The first and third quartiles (25% and 75%) are indicated as 1Q and 3Q. Analyses were performed in the R statistical language (R 3.2.1).

### Repertoire statistics

For each sample, TCR repertoires were characterized by their relative frequency, (p1 > p2 > … > pN) and for clarity we have referred to entropy (Shannon’s entropy) $H\left(p\right)=\sum_{k}p_{k}\log_{2}p\_k$ and clonality (1-Pielou’s index): $C\left(p\right)=1-\frac{H\left(p\right)}{\log_{2}N}$. TOP1 refers to the relative frequency of the most common clone and N25 is the minimum number of clones required to capture 25% of the repertore $N25=\min\left\{k:\sum_{j=1}^{k}p_{\left(j\right)}>0.25\right\}$. Other statistics are introduced in text.

### Study approval

Written informed consent was received from study subjects under an Institutional Review Board (IRB) protocol CIC 02-15.

## SUPPLEMENTARY MATERIALS





## Abbreviations

CDR: complementarity determining region
CT: cancer testis
HR: hazard ratio
IHC: immunohistochemistry
OS: overall survival
PBMC: peripheral blood mononuclear cell
PFS: progression free survival
TAA: tumor associated antigen
TCGA: The Cancer Genome Atlas
TCR: T cell receptor
TIL: tumor infiltrating lymphocyte
