# Supplementary material for: Clonality and antigen-specific responses shape the prognostic effects of tumor-infiltrating T cells in ovarian cancer
Source: Oncotarget. 2020 Jul 7;11(27):2669–83. doi: 10.18632/oncotarget.27666 (PMC7343634; doi:10.18632/oncotarget.27666)
Supplement: Supplementary file 1 [file oncotarget-11-2669-s001.pdf]

Clonality and antigen-specific responses shape the prognostic effects of tumor-infiltrating T cells in ovarian cancer

SUPPLEMENTARY MATERIALS

Statistical methods

Examining the distribution of clone frequencies, we used Fisher's model for unseen species assuming an exponential model (shape = 1) to calculate the sequencing coverage; we estimated that 78% of tumor samples have at least 90% coverage and the median coverage is 83%. Low coverage was not associated with any of the repertoire statistics.

Supplementary Figure 1 and Supplementary Table 2 contain a spread of statistics meant to capture different features of the repertoire. We report on clonality as defined above as well as the TOPX statistics (the % of the repertoire captured by the most common X reads), the NX statistics (the number of clones required to capture X% of the repertoire) and the RX statistics (the % of the PBMC repertoire that maps to the corresponding clones identified by NX).

Patient characteristics and survival data

Study outcomes included overall survival (OS), the time until death; and progression-free survival (PFS), the time to recurrence or progression, both measured from the time of definitive surgery. Progression or recurrence was defined by time to radiologically confirmed relapse of disease. If no imaging data were available, progression was determined by increase in CA125 to twice the upper limit of normal or two times the nadir value if CA125 was never normal during primary treatment.

Sequencing variation and TCR repertoire statistics depend on degree of infiltration

Each patient's TIL TCR repertoire contained between 224 and 94,770 (1Q: 2,918; 3Q 15,190; median: 11,590) unique AA sequences (Supplementary Table 2). The number of reads in the tumor specimens (17,978–13,368,648) varied over three orders of magnitude and was associated with density of $CD3^+$ T cell infiltration measured by IHC (Spearman's rho = 0.340, $p = 0.001$). Between 65.3% (1st quartile) and 84.0% (3rd quartile) of reads are captured by the most common 1000 clones implying that most samples have a large number of rare reads.

To leverage the deep TCR sequence and frequency information in our 99 samples, we first analyzed the variety of TCR sequences across all patients. We obtained 992,791 unique TIL TCR sequences in 289,963,375 normalized reads in combined 99 patients. In matched PBMC samples, there were 6,275,193 unique clones in 400,143,279 normalized reads. We found that, on average, two patients share 2.6% of PBMC clones (range: 0.5–4.7%) which is lower than previously reported results.

Noting that there were similar overall levels of reads, the overall TIL repertoire appeared more restricted than the PBMC repertoire. The most shared TIL clone was found in only 41 of the 99 patients while the most shared PBMC clones appeared in more than 90 of the patients. Again these clones did not comprise an unusually large fraction of the reads.

With respect to the usage of specific clones in the repertoire, the average rate of reads to their unique TCR clone may describe the evenness. For example, the mean ovarian cancer TIL repertoire had one unique clone for every 364 reads (median 1 per 199 reads). The range varied from 1 per 2714 reads (strongly restricted) to 1 per 25 reads, which was close to the noise threshold at 20 reads.

Individual TIL repertoires

We describe each TIL repertoire by plotting the relative frequency of each clone in order from most common to least using the log-rank to emphasize the most common clone and to de-emphasize the long tail of low abundance clones. All the plots in Supplementary Figure 2 have the same x-axis so the longer the right tail, the more unique clones in the repertoire. Additionally, we have split the cohort into roughly equal sized bins based on the TOP1 statistic. Therefore the more monoclonal cases and the more polyclonal cases are grouped together and the set of putatively oligoclonal cases are in between. Repertoire specific statistics are given for each patient.

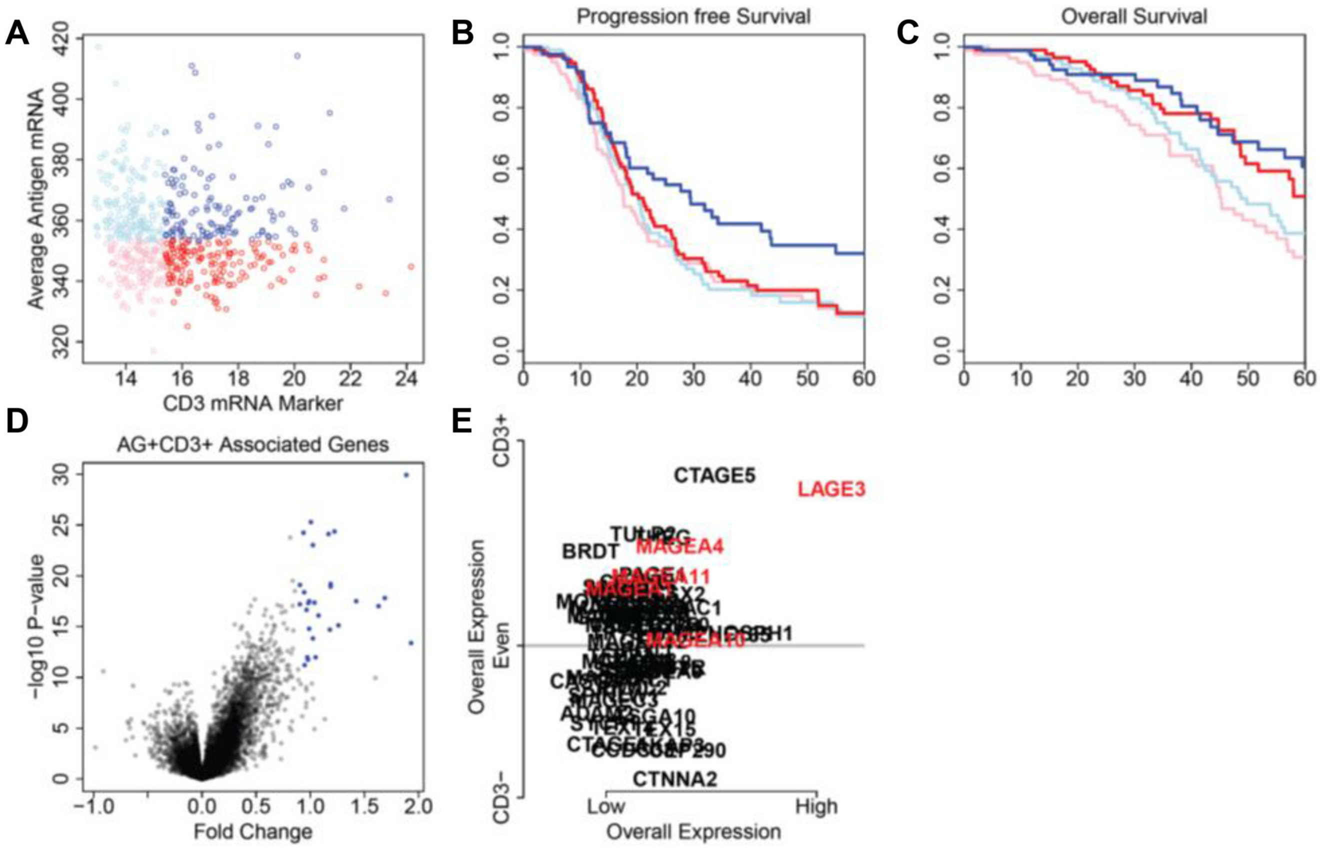

Supplementary Figure 1: Prognostic effect of T cell infiltration and CT antigen levels in TCGA cohort. (**A**) mRNA expression of T cell markers (CD3, CD8, GZMB, IL2) and 91 unselected CT antigens used to form an antigen burden score. Median expression levels used to stratify into 4 groups based on CD3 levels and CT antigen burden. Prognostic effect of CD3 infiltration and CT antigen burden for (**B**) PFS and (**C**) and OS. (**D**) Volcano plot of gene levels in $CD3^{+}$CT $antigen^{hi}$ category. Upregulated genes in blue include CXCL9/10, IFI6/35 and ISG15 (interferon induced). (**E**) Composite analysis of CT antigen levels based on overall CD3 T cell infiltration.

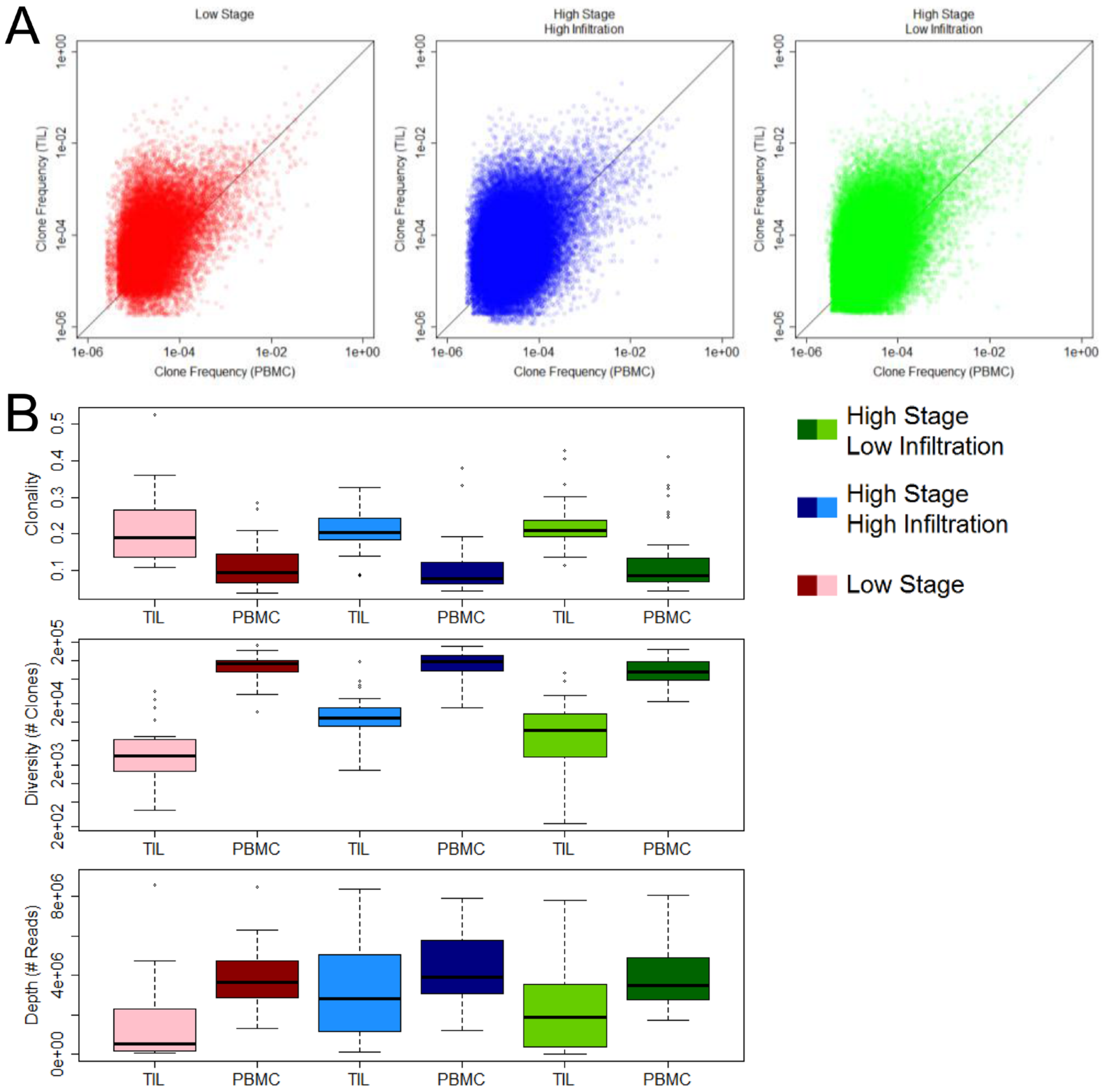

Supplementary Figure 2: Overview of TCR sequencing results. (**A**) 4.91 million clones from 99 ovarian cancer patients show a wide variety of clone frequency between the tumor infiltrating (TIL) and peripheral repertoires (PBMC) across stage and degree of infiltration. (**B**) Clonality and diversity summarize variation in the TCR repertoire; depth is a measure of sequencing quality and observable TCR sequences in samples.

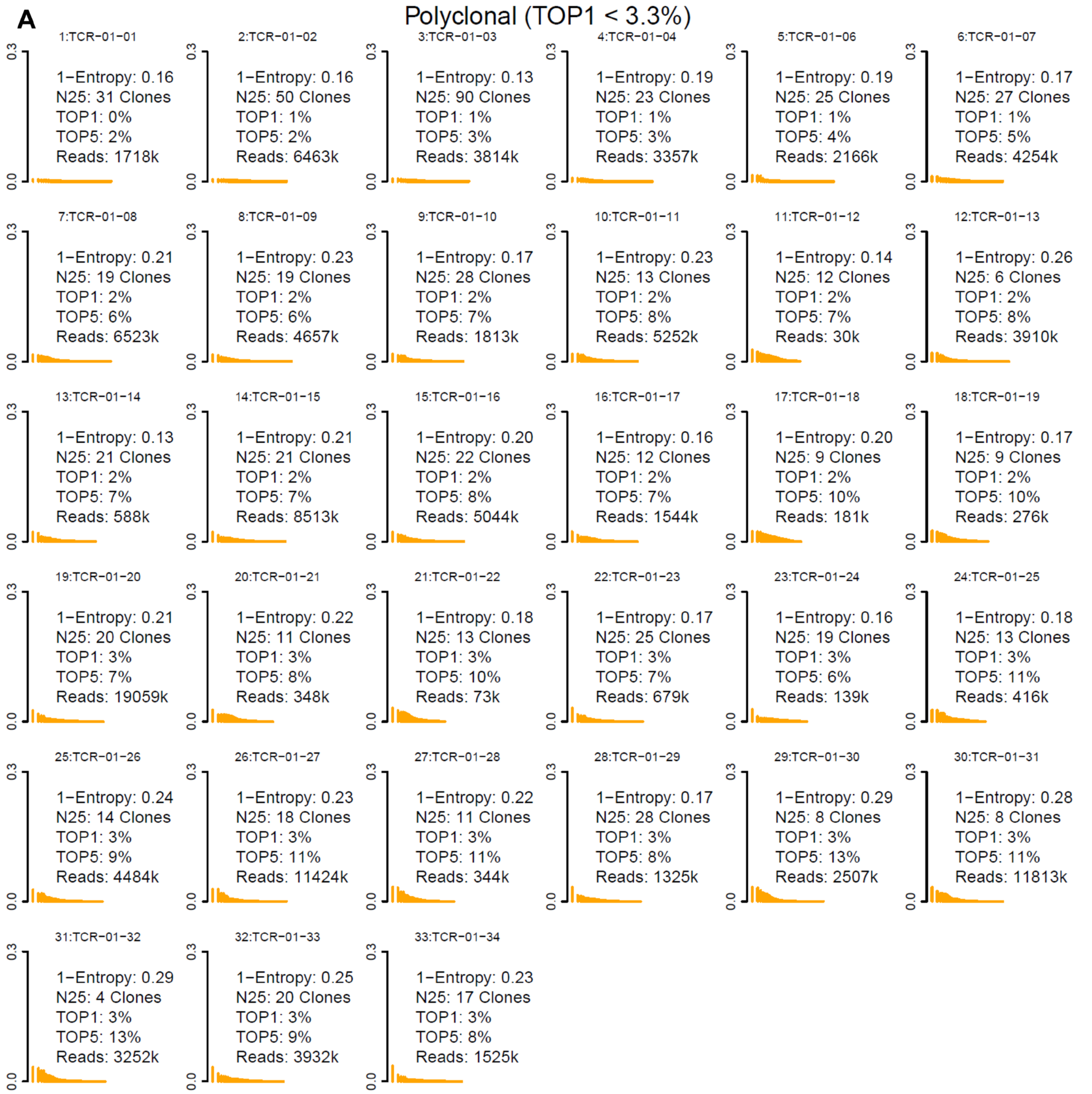
A
Polyclonal (TOP1 < 3.3%)
1:TCR−01−01
1−Entropy: 0.16
N25: 31 Clones
TOP1: 0%
TOP5: 2%
Reads: 1718k
2:TCR−01−02
1−Entropy: 0.16
N25: 50 Clones
TOP1: 1%
TOP5: 2%
Reads: 6463k
3:TCR−01−03
1−Entropy: 0.13
N25: 90 Clones
TOP1: 1%
TOP5: 3%
Reads: 3814k
4:TCR−01−04
1−Entropy: 0.19
N25: 23 Clones
TOP1: 1%
TOP5: 3%
Reads: 3357k
5:TCR−01−06
1−Entropy: 0.19
N25: 25 Clones
TOP1: 1%
TOP5: 4%
Reads: 2166k
6:TCR−01−07
1−Entropy: 0.17
N25: 27 Clones
TOP1: 1%
TOP5: 5%
Reads: 4254k
7:TCR−01−08
1−Entropy: 0.21
N25: 19 Clones
TOP1: 2%
TOP5: 6%
Reads: 6523k
8:TCR−01−09
1−Entropy: 0.23
N25: 19 Clones
TOP1: 2%
TOP5: 6%
Reads: 4657k
9:TCR−01−10
1−Entropy: 0.17
N25: 28 Clones
TOP1: 2%
TOP5: 7%
Reads: 1813k
10:TCR−01−11
1−Entropy: 0.23
N25: 13 Clones
TOP1: 2%
TOP5: 8%
Reads: 5252k
11:TCR−01−12
1−Entropy: 0.14
N25: 12 Clones
TOP1: 2%
TOP5: 7%
Reads: 30k
12:TCR−01−13
1−Entropy: 0.26
N25: 6 Clones
TOP1: 2%
TOP5: 8%
Reads: 3910k
13:TCR−01−14
1−Entropy: 0.13
N25: 21 Clones
TOP1: 2%
TOP5: 7%
Reads: 588k
14:TCR−01−15
1−Entropy: 0.21
N25: 21 Clones
TOP1: 2%
TOP5: 7%
Reads: 8513k
15:TCR−01−16
1−Entropy: 0.20
N25: 22 Clones
TOP1: 2%
TOP5: 8%
Reads: 5044k
16:TCR−01−17
1−Entropy: 0.16
N25: 12 Clones
TOP1: 2%
TOP5: 7%
Reads: 1544k
17:TCR−01−18
1−Entropy: 0.20
N25: 9 Clones
TOP1: 2%
TOP5: 10%
Reads: 181k
18:TCR−01−19
1−Entropy: 0.17
N25: 9 Clones
TOP1: 2%
TOP5: 10%
Reads: 276k
19:TCR−01−20
1−Entropy: 0.21
N25: 20 Clones
TOP1: 3%
TOP5: 7%
Reads: 19059k
20:TCR−01−21
1−Entropy: 0.22
N25: 11 Clones
TOP1: 3%
TOP5: 8%
Reads: 348k
21:TCR−01−22
1−Entropy: 0.18
N25: 13 Clones
TOP1: 3%
TOP5: 10%
Reads: 73k
22:TCR−01−23
1−Entropy: 0.17
N25: 25 Clones
TOP1: 3%
TOP5: 7%
Reads: 679k
23:TCR−01−24
1−Entropy: 0.16
N25: 19 Clones
TOP1: 3%
TOP5: 6%
Reads: 139k
24:TCR−01−25
1−Entropy: 0.18
N25: 13 Clones
TOP1: 3%
TOP5: 11%
Reads: 416k
25:TCR−01−26
1−Entropy: 0.24
N25: 14 Clones
TOP1: 3%
TOP5: 9%
Reads: 4484k
26:TCR−01−27
1−Entropy: 0.23
N25: 18 Clones
TOP1: 3%
TOP5: 11%
Reads: 11424k
27:TCR−01−28
1−Entropy: 0.22
N25: 11 Clones
TOP1: 3%
TOP5: 11%
Reads: 344k
28:TCR−01−29
1−Entropy: 0.17
N25: 28 Clones
TOP1: 3%
TOP5: 8%
Reads: 1325k
29:TCR−01−30
1−Entropy: 0.29
N25: 8 Clones
TOP1: 3%
TOP5: 13%
Reads: 2507k
30:TCR−01−31
1−Entropy: 0.28
N25: 8 Clones
TOP1: 3%
TOP5: 11%
Reads: 11813k
31:TCR−01−32
1−Entropy: 0.29
N25: 4 Clones
TOP1: 3%
TOP5: 13%
Reads: 3252k
32:TCR−01−33
1−Entropy: 0.25
N25: 20 Clones
TOP1: 3%
TOP5: 9%
Reads: 3932k
33:TCR−01−34
1−Entropy: 0.23
N25: 17 Clones
TOP1: 3%
TOP5: 8%
Reads: 1525k

B

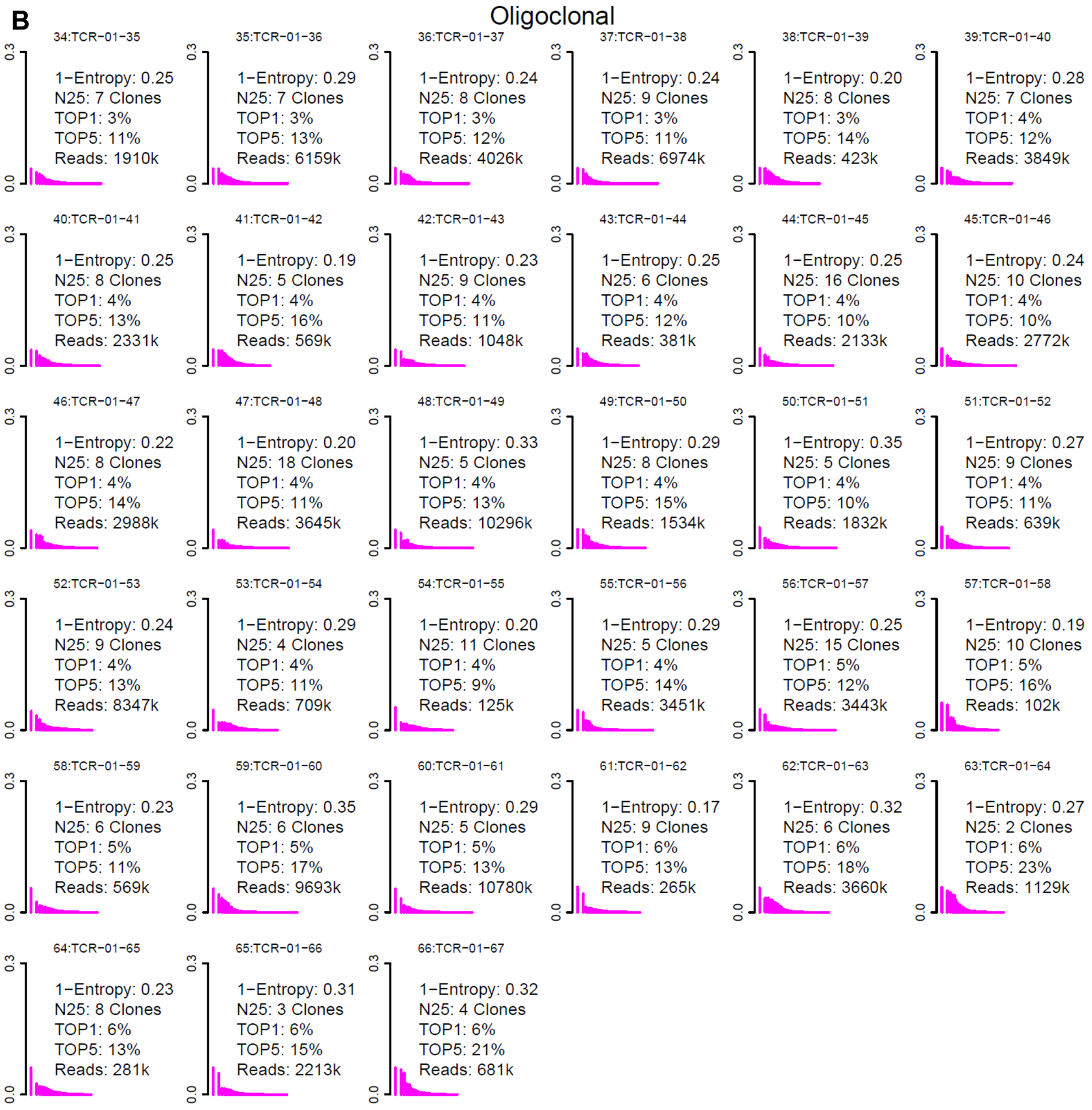
Oligoclonal
34:TCR−01−35
1−Entropy: 0.25
N25: 7 Clones
TOP1: 3%
TOP5: 11%
Reads: 1910k
35:TCR−01−36
1−Entropy: 0.29
N25: 7 Clones
TOP1: 3%
TOP5: 13%
Reads: 6159k
36:TCR−01−37
1−Entropy: 0.24
N25: 8 Clones
TOP1: 3%
TOP5: 12%
Reads: 4026k
37:TCR−01−38
1−Entropy: 0.24
N25: 9 Clones
TOP1: 3%
TOP5: 11%
Reads: 6974k
38:TCR−01−39
1−Entropy: 0.20
N25: 8 Clones
TOP1: 3%
TOP5: 14%
Reads: 423k
39:TCR−01−40
1−Entropy: 0.28
N25: 7 Clones
TOP1: 4%
TOP5: 12%
Reads: 3849k
40:TCR−01−41
1−Entropy: 0.25
N25: 8 Clones
TOP1: 4%
TOP5: 13%
Reads: 2331k
41:TCR−01−42
1−Entropy: 0.19
N25: 5 Clones
TOP1: 4%
TOP5: 16%
Reads: 569k
42:TCR−01−43
1−Entropy: 0.23
N25: 9 Clones
TOP1: 4%
TOP5: 11%
Reads: 1048k
43:TCR−01−44
1−Entropy: 0.25
N25: 6 Clones
TOP1: 4%
TOP5: 12%
Reads: 381k
44:TCR−01−45
1−Entropy: 0.25
N25: 16 Clones
TOP1: 4%
TOP5: 10%
Reads: 2133k
45:TCR−01−46
1−Entropy: 0.24
N25: 10 Clones
TOP1: 4%
TOP5: 10%
Reads: 2772k
46:TCR−01−47
1−Entropy: 0.22
N25: 8 Clones
TOP1: 4%
TOP5: 14%
Reads: 2988k
47:TCR−01−48
1−Entropy: 0.20
N25: 18 Clones
TOP1: 4%
TOP5: 11%
Reads: 3645k
48:TCR−01−49
1−Entropy: 0.33
N25: 5 Clones
TOP1: 4%
TOP5: 13%
Reads: 10296k
49:TCR−01−50
1−Entropy: 0.29
N25: 8 Clones
TOP1: 4%
TOP5: 15%
Reads: 1534k
50:TCR−01−51
1−Entropy: 0.35
N25: 5 Clones
TOP1: 4%
TOP5: 10%
Reads: 1832k
51:TCR−01−52
1−Entropy: 0.27
N25: 9 Clones
TOP1: 4%
TOP5: 11%
Reads: 639k
52:TCR−01−53
1−Entropy: 0.24
N25: 9 Clones
TOP1: 4%
TOP5: 13%
Reads: 8347k
53:TCR−01−54
1−Entropy: 0.29
N25: 4 Clones
TOP1: 4%
TOP5: 11%
Reads: 709k
54:TCR−01−55
1−Entropy: 0.20
N25: 11 Clones
TOP1: 4%
TOP5: 9%
Reads: 125k
55:TCR−01−56
1−Entropy: 0.29
N25: 5 Clones
TOP1: 4%
TOP5: 14%
Reads: 3451k
56:TCR−01−57
1−Entropy: 0.25
N25: 15 Clones
TOP1: 5%
TOP5: 12%
Reads: 3443k
57:TCR−01−58
1−Entropy: 0.19
N25: 10 Clones
TOP1: 5%
TOP5: 16%
Reads: 102k
58:TCR−01−59
1−Entropy: 0.23
N25: 6 Clones
TOP1: 5%
TOP5: 11%
Reads: 569k
59:TCR−01−60
1−Entropy: 0.35
N25: 6 Clones
TOP1: 5%
TOP5: 17%
Reads: 9693k
60:TCR−01−61
1−Entropy: 0.29
N25: 5 Clones
TOP1: 5%
TOP5: 13%
Reads: 10780k
61:TCR−01−62
1−Entropy: 0.17
N25: 9 Clones
TOP1: 6%
TOP5: 13%
Reads: 265k
62:TCR−01−63
1−Entropy: 0.32
N25: 6 Clones
TOP1: 6%
TOP5: 18%
Reads: 3660k
63:TCR−01−64
1−Entropy: 0.27
N25: 2 Clones
TOP1: 6%
TOP5: 23%
Reads: 1129k
64:TCR−01−65
1−Entropy: 0.23
N25: 8 Clones
TOP1: 6%
TOP5: 13%
Reads: 281k
65:TCR−01−66
1−Entropy: 0.31
N25: 3 Clones
TOP1: 6%
TOP5: 15%
Reads: 2213k
66:TCR−01−67
1−Entropy: 0.32
N25: 4 Clones
TOP1: 6%
TOP5: 21%
Reads: 681k
0.3
0.0

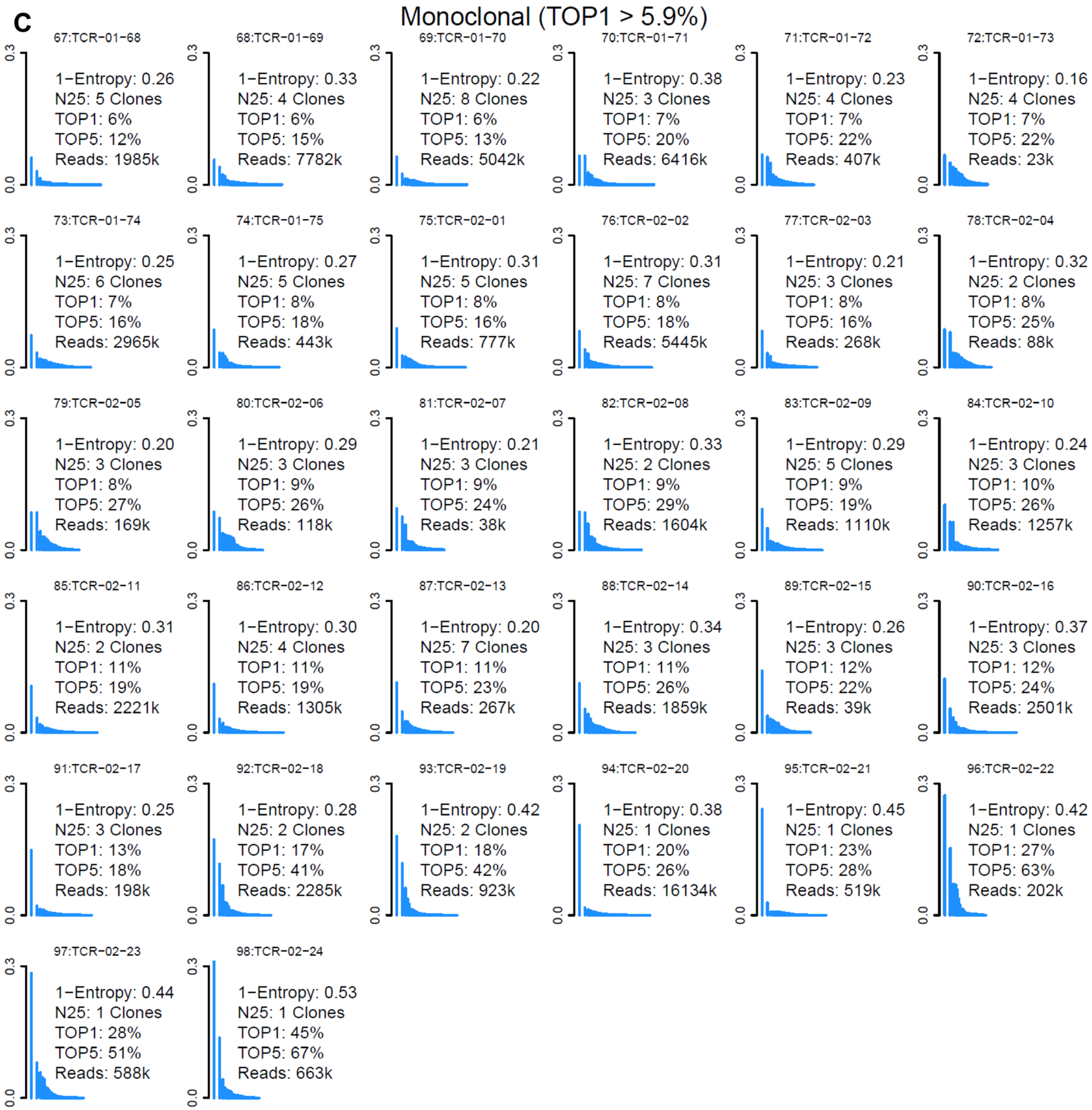

Supplementary Figure 3: Individual patient TCR repertoire composition. TCR repertoire can be stratified into three categories: (**A**) polyclonal, (**B**) oligoclonal, or (**C**) monoclonal.

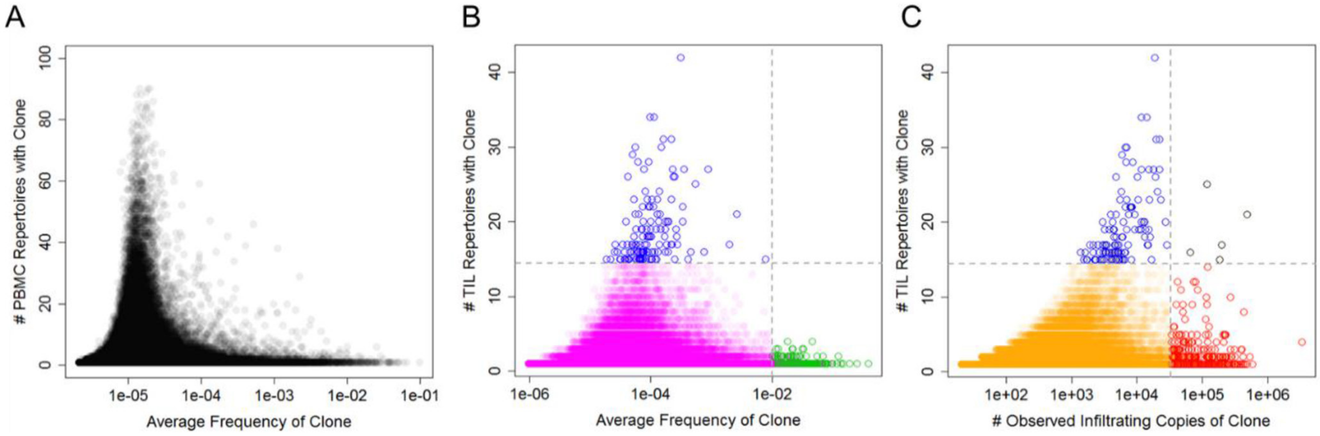

Supplementary Figure 4: TCR clones common to multiple repertoires. Clones sharing and frequency in PBMC samples (**A**) and across TIL repertoires versus their average frequency (**B**) or depth of infiltration (**C**). Note that the scale of the y-axes differs between plots. Clone subsets are highlighted if they are common to multiple repertoires (blue), relatively frequent (green) or have a high level of absolute infiltration (red).

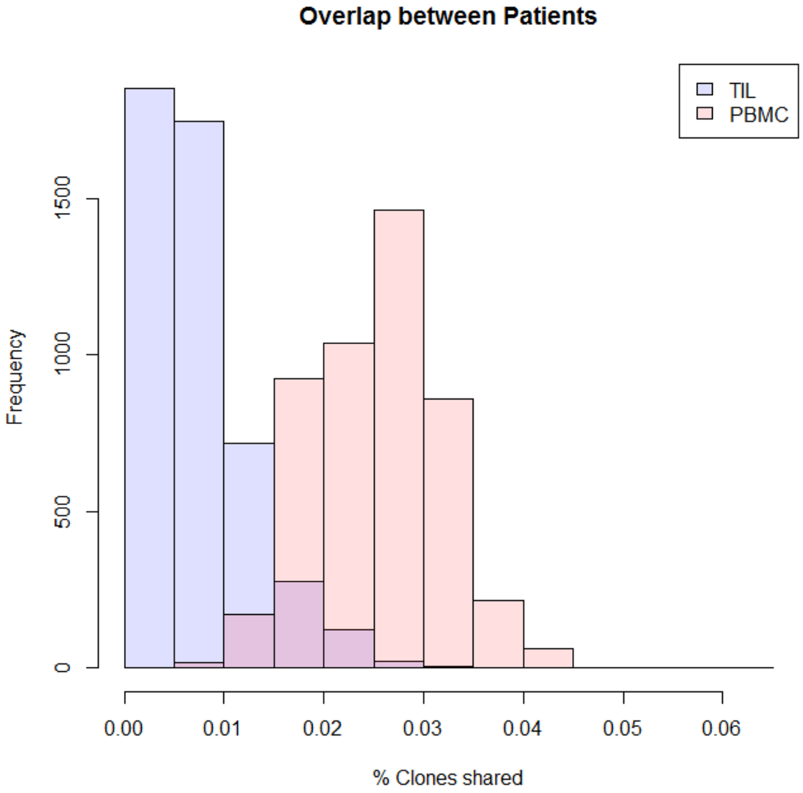

Supplementary Figure 5: Clone overlap between TIL and PBMC repertoires. Overlap between patient repertoires is tissue specific: TIL repertoires are more exclusive than PBMC repertoires.

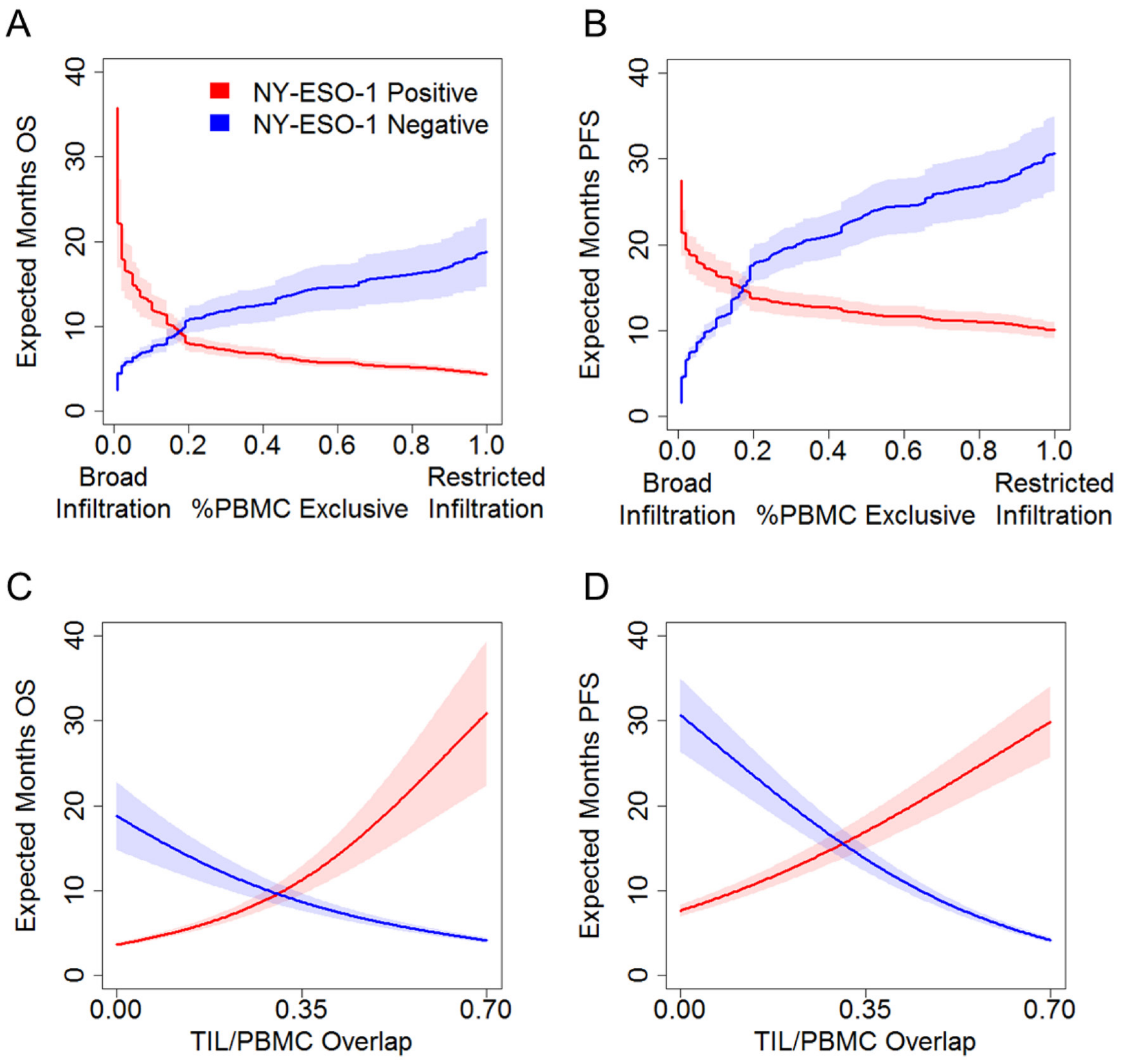

Supplementary Figure 6: Restricted mean survival (RMS) overall survival and progression-free survival estimates for the effect of exclusivity and clone-level overlap between TIL and PBMC repertoires. Cox model-based RMS estimates for PBMC exclusivity stratified by NY-ESO-1 serology for OS (**A**) and PFS (**B**); clone overlap stratified on NY-ESO-1 serology (**C**, **D**).

Supplementary Table 1: Prognostic features of TCR repertoires across studies

	Overall Survival		Progression Free Survival	
	HR	*p*-value	HR	*p*-value
TCGA-TIL				
Entropy	0.76 (0.64–0.91)	0.003209	0.86 (0.74–1.01)	0.05778
Clonality	1.07 (0.89–1.30)	0.462697	1.08 (0.92–1.26)	0.37364
Infiltration	0.78 (0.65–0.94)	0.008096	0.86 (0.74–1.01)	0.05753
Diversity	0.77 (0.64–0.92)	0.003699	0.87 (0.75–1.01)	0.05925
TOP1	1.36 (1.14–1.64)	0.000807	1.19 (1.02–1.38)	0.02547
N25	0.72 (0.58–0.90)	0.002949	0.80 (0.68–0.95)	0.00932
RPCI-TIL				
Entropy	0.78 (0.59–1.04)	0.0932	0.95 (0.75–1.21)	0.699
Clonality	1.21 (0.90–1.62)	0.2032	0.99 (0.76–1.30)	0.9528
Infiltration	0.91 (0.68–1.21)	0.5065	0.81 (0.64–1.03)	0.0901
Diversity	0.82 (0.62–1.09)	0.1766	0.97 (0.75–1.23)	0.7763
TOP1	1.13 (0.84–1.51)	0.4149	1.04 (0.80–1.35)	0.7765
N25	0.81 (0.54–1.22)	0.3182	0.84 (0.6–1.19)	0.3299
RPCI-PBMC				
Entropy	0.79 (0.61–1.03)	0.081	0.87 (0.68–1.11)	0.2686
Clonality	1.20 (0.92–1.58)	0.1821	1.09 (0.84–1.43)	0.513
Infiltration	0.84 (0.65–1.09)	0.194	0.84 (0.69–1.02)	0.0802
Diversity	0.80 (0.62–1.03)	0.0832	0.84 (0.67–1.06)	0.14
TOP1	1.19 (0.92–1.52)	0.1804	1.02 (0.78–1.35)	0.8711
N25	1.11 (0.87–1.42)	0.4135	0.97 (0.78–1.22)	0.8216

Supplementary Table 2: TCR sequencing on matched TIL and PBMC samples from RPCI ovarian cancer cohort. See Supplementary Table 2

Supplementary Table 3: TCR clones and read processing

TIL	Total Clones	Unique Clones	Observed Reads	Normalized Reads
Observed NT sequences	1,454,072	1,443,896	305,389,678	347,052,958
Productive NT sequences	1,194,008	1,184,830	259,152,997	289,963,375
Unique AA sequences	1,136,217	992,791	259,152,997	289,963,375
Sequence found in PBMC	243,510	213,072	141,510,702	152,888,787
% TIL Exclusive	78.56%	78.53%	45.39%	47.27%
**PBMC**	**Total Clones**	**Unique Clones**	**Observed Reads**	**Normalized Reads**
Observed	10,487,218	10,337,157	505,261,517	479,220,568
Productive	8,785,710	8,637,309	400,143,279	432,017,588
AA Unique	8,213,806	6,275,193	432,017,588	400,143,279

NT: Nucleotide, AA: Amino Acid.

Supplementary Table 4: Association between the top 18 common clones and PFS

		Frequency of clone			Log frequency of clone			Frequency Rank clone		
Clone	#subjects with clone	log HR	*p*-value	Bonferroni *p*	log HR	*p*-value	Bonferroni *p*	log HR	*p*-value	Bonferroni *p*
CASSLGETQYF	42	0.015	0.893	1.000	–0.033	0.784	1.000	–0.035	0.771	1.000
CASSLQETQYF	34	–0.210	0.231	1.000	–0.150	0.222	1.000	–0.151	0.222	1.000
CASSLGGNTEAFF	34	0.038	0.722	1.000	–0.016	0.893	1.000	–0.017	0.885	1.000
**CASSLTDTQYF**	**31**	**0.428**	**0.003**	**0.046**	**0.050**	**0.668**	**1.000**	**0.036**	**0.757**	**1.000**
CASSLSTDTQYF	31	–0.104	0.360	1.000	–0.128	0.278	1.000	–0.131	0.273	1.000
CASSFQETQYF	30	–0.152	0.385	1.000	–0.087	0.472	1.000	–0.085	0.484	1.000
CASSLGYEQYF	30	–0.077	0.496	1.000	–0.010	0.928	1.000	–0.017	0.881	1.000
CASSSQETQYF	29	–0.074	0.517	1.000	–0.028	0.810	1.000	–0.029	0.802	1.000
CASSLGTEAFF	28	0.008	0.946	1.000	–0.097	0.416	1.000	–0.102	0.393	1.000
CASSSTDTQYF	28	–0.240	0.290	1.000	0.035	0.760	1.000	0.030	0.795	1.000
CASSPSTDTQYF	27	–0.059	0.568	1.000	–0.101	0.389	1.000	–0.095	0.421	1.000
CASSLNTEAFF	27	–0.075	0.684	1.000	0.169	0.145	1.000	0.178	0.126	1.000
CASSLSYEQYF	27	0.250	0.036	0.649	0.114	0.326	1.000	0.103	0.368	1.000
CASSLGPNTEAFF	27	0.136	0.161	1.000	0.230	0.049	0.873	0.225	0.054	0.975
CASSSSTDTQYF	26	0.151	0.169	1.000	0.105	0.368	1.000	0.099	0.393	1.000
CASSLRETQYF	26	0.027	0.787	1.000	0.157	0.164	1.000	0.163	0.152	1.000
CASSFLREPNYGYTF	26	–0.107	0.449	1.000	–0.077	0.510	1.000	–0.067	0.568	1.000
CASSLGRNTEAFF	25	–0.438	0.687	1.000	0.079	0.502	1.000	0.099	0.399	1.000

← Only Bonferroni significant clone.

Supplementary Table 5: Cox model regression stratified by stage

	Overall Survival	Progression-Free Survival
**PBMC Exclusivity (Stage Stratified)**	**HR (95%CI), score test *p*-value**	**HR (95%CI), score test *p*-value**
Exclusivity*	0.06 (0.00–0.61), 0.017	0.70 (0.47–1.03), 0.068
NY-ESO-1 Status	1.68 (0.94–2.99), 0.078	1.83 (1.08–3.10), 0.026
Log10 CD3 level*	0.68 (0.52–0.88), 0.003	0.79 (0.34–0.99), 0.037
Overlap vs NYESO-1	1.91 (1.08–3.36), 0.025	2.17 (1.18–3.99), 0.013
Overall model fit	Wald Test $p = 0.003$	Wald Test $p = 0.011$
Deviance test (Interaction)	$p = 0.022$	$p = 0.011$

	Overall Survival	Progression-Free Survival
**TIL/PBMC Overlap (Stage Stratified)**	**HR (95%CI), score test *p*-value**	**HR (95%CI), score test *p*-value**
Overlap*	1.51 (1.08–2.12), 0.015	1.39 (0.99–1.96), 0.058
NY-ESO-1 Status	1.67 (0.95–2.95), 0.078	1.78 (1.06–3.00), 0.030
Log10 CD3 level*	0.66 (0.51–0.86), 0.002	0.77 (0.62–0.96), 0.021
Overlap vs NYESO-1	0.49 (0.27–0.87), 0.016	0.44 (0.25–0.76), 0.004
Overall model fit	Wald Test $p = 0.003$	Wald Test $p = 0.004$
Deviance test (Interaction)	$p = 0.016$	$p = 0.004$

*Scaled to unit std. deviation.
