# Supplementary material for: Clonality and antigen-specific responses shape the prognostic effects of tumor-infiltrating T cells in ovarian cancer
Source: Oncotarget. 2020 Jul 7;11(27):2669–83. doi: 10.18632/oncotarget.27666 (PMC7343634; doi:10.18632/oncotarget.27666)
Supplement: Supplementary file 2 [file oncotarget-11-2669-s002.docx]

| **Supplementary Table 2: TCR sequencing on matched TIL and PBMC samples from RPCI ovarian cancer cohort** | | | | | | | | | | | | | | | | | |
| --- | --- | --- | --- | --- | --- | --- | --- | --- | --- | --- | --- | --- | --- | --- | --- | --- | --- |
|  |  | All | ESO‒ | ESO+ | pvalue |  |  | EXP‒ | EXP+ | SEROPOS |  |  | NEG | MAGE1 | MAGE3 | P53 | NYESO1 |
| n |  | 98.00 | 74.00 | 25.00 |  |  |  | 54.00 | 20.00 | 25.00 |  |  | 61.00 | 2.00 | 2.00 | 9.00 | 25.00 |
| NY-ESO-1 | % Positive | 0.25 | 0.00 | 1.00 |  |  |  | 0.00 | 0.00 | 1.00 |  |  | 0.00 | 0.00 | 0.00 | 0.00 | 1.00 |
| LAGE-1 | % Positive | 0.21 | 0.00 | 0.80 |  |  |  | 0.00 | 0.00 | 0.80 |  |  | 0.00 | 0.00 | 0.00 | 0.00 | 0.80 |
| MAGE-1 | % Positive | 0.05 | 0.03 | 0.12 |  |  |  | 0.04 | 0.00 | 0.12 |  |  | 0.00 | 1.00 | 0.00 | 0.00 | 0.12 |
| MAGE-3 | % Positive | 0.07 | 0.03 | 0.20 |  |  |  | 0.02 | 0.05 | 0.20 |  |  | 0.00 | 0.00 | 1.00 | 0.00 | 0.20 |
| P53 | % Positive | 0.17 | 0.13 | 0.28 |  |  |  | 0.14 | 0.10 | 0.28 |  |  | 0.02 | 0.00 | 0.00 | 0.89 | 0.28 |
| DHFR | % Positive | 0.02 | 0.00 | 0.09 |  |  |  | 0.00 | 0.00 | 0.09 |  |  | 0.00 | 0.00 | 0.00 | 0.00 | 0.09 |
| Tumor | Clones | 11594.05 | 11287.96 | 12537.83 | 0.67 |  |  | 12564.33 | 7841.75 | 12537.83 |  |  | 12053.92 | 2929.00 | 2264.00 | 9959.33 | 12537.83 |
|  | Normalized Reads | 2958809.95 | 2481820.14 | 4429528.54 | 0.06 |  |  | 2342009.69 | 2859308.35 | 4429528.54 |  |  | 2236965.05 | 4393718.00 | 485405.00 | 4160175.11 | 4429528.54 |
|  | Observed Reads | 2644418.34 | 2185461.39 | 4059535.58 | 0.02 |  |  | 2163504.59 | 2244744.75 | 4059535.58 |  |  | 2091385.00 | 3074806.00 | 570082.00 | 2984431.33 | 4059535.58 |
|  | % less than 20 copies | 0.33 | 0.36 | 0.27 | 0.00 |  |  | 0.36 | 0.36 | 0.27 |  |  | 0.35 | 0.30 | 0.50 | 0.36 | 0.27 |
|  | Clonality (1-entropy) | 0.22 | 0.21 | 0.23 | 0.48 |  |  | 0.21 | 0.22 | 0.23 |  |  | 0.21 | 0.21 | 0.24 | 0.23 | 0.23 |
|  | Clonality (PBMC overlap) | 0.25 | 0.25 | 0.26 | 0.46 |  |  | 0.25 | 0.26 | 0.26 |  |  | 0.25 | 0.22 | 0.28 | 0.27 | 0.26 |
| PBMC | Clones | 83814.35 | 87593.30 | 72162.58 | 0.11 |  |  | 83724.83 | 98038.15 | 72162.58 |  |  | 86781.93 | 66141.50 | 96144.50 | 95959.33 | 72162.58 |
|  | Normalized Reads | 4083094.68 | 4283908.28 | 3463919.42 | 0.01 |  |  | 4377701.76 | 4030665.90 | 3463919.42 |  |  | 4343269.31 | 3298984.50 | 3391531.00 | 4298750.44 | 3463919.42 |
|  | Observed Reads | 4408342.73 | 4615623.04 | 3769228.46 | 0.01 |  |  | 4728951.54 | 4309636.10 | 3769228.46 |  |  | 4686529.13 | 3358358.50 | 3807479.00 | 4594017.00 | 3769228.46 |
|  | % less than 20 copies | 0.44 | 0.45 | 0.39 | 0.11 |  |  | 0.44 | 0.48 | 0.39 |  |  | 0.44 | 0.54 | 0.58 | 0.49 | 0.39 |
|  | Clonality (1-entropy) | 0.12 | 0.11 | 0.13 | 0.43 |  |  | 0.11 | 0.11 | 0.13 |  |  | 0.11 | 0.21 | 0.14 | 0.12 | 0.13 |
|  | Clonality (PBMC overlap) | 0.30 | 0.30 | 0.31 | 0.77 |  |  | 0.30 | 0.31 | 0.31 |  |  | 0.29 | 0.49 | 0.42 | 0.30 | 0.31 |
| Tumor | TOP1 (Freq. of most common clone) | 0.06 | 0.06 | 0.06 | 0.98 |  |  | 0.06 | 0.07 | 0.06 |  |  | 0.06 | 0.04 | 0.10 | 0.07 | 0.06 |
|  | TOP5 | 0.16 | 0.15 | 0.16 | 0.91 |  |  | 0.15 | 0.18 | 0.16 |  |  | 0.15 | 0.13 | 0.25 | 0.16 | 0.16 |
|  | TOP10 | 0.21 | 0.21 | 0.21 | 0.93 |  |  | 0.20 | 0.24 | 0.21 |  |  | 0.20 | 0.19 | 0.30 | 0.21 | 0.21 |
|  | TOP20 | 0.27 | 0.27 | 0.28 | 0.89 |  |  | 0.26 | 0.30 | 0.28 |  |  | 0.27 | 0.26 | 0.37 | 0.28 | 0.28 |
|  | TOP25 | 0.29 | 0.29 | 0.30 | 0.87 |  |  | 0.28 | 0.33 | 0.30 |  |  | 0.29 | 0.28 | 0.40 | 0.30 | 0.30 |
|  | TOP30 | 0.31 | 0.31 | 0.32 | 0.86 |  |  | 0.30 | 0.35 | 0.32 |  |  | 0.31 | 0.30 | 0.42 | 0.32 | 0.32 |
|  | TOP40 | 0.34 | 0.34 | 0.35 | 0.84 |  |  | 0.33 | 0.38 | 0.35 |  |  | 0.34 | 0.34 | 0.45 | 0.36 | 0.35 |
|  | TOP50 | 0.37 | 0.37 | 0.38 | 0.82 |  |  | 0.35 | 0.41 | 0.38 |  |  | 0.36 | 0.37 | 0.47 | 0.38 | 0.38 |
|  | TOP100 | 0.45 | 0.45 | 0.46 | 0.77 |  |  | 0.44 | 0.49 | 0.46 |  |  | 0.44 | 0.47 | 0.55 | 0.48 | 0.46 |
|  | TOP200 | 0.55 | 0.54 | 0.56 | 0.71 |  |  | 0.53 | 0.58 | 0.56 |  |  | 0.53 | 0.61 | 0.64 | 0.58 | 0.56 |
|  | TOP500 | 0.66 | 0.66 | 0.67 | 0.75 |  |  | 0.65 | 0.68 | 0.67 |  |  | 0.64 | 0.82 | 0.79 | 0.71 | 0.67 |
|  | TOP1000 | 0.73 | 0.74 | 0.71 | 0.42 |  |  | 0.72 | 0.80 | 0.71 |  |  | 0.73 | 0.93 | 0.92 | 0.74 | 0.71 |
| PBMC | TOP1 (Freq. of most common clone) | 0.03 | 0.03 | 0.05 | 0.28 |  |  | 0.03 | 0.03 | 0.05 |  |  | 0.03 | 0.06 | 0.05 | 0.02 | 0.05 |
|  | TOP5 | 0.08 | 0.07 | 0.10 | 0.25 |  |  | 0.07 | 0.07 | 0.10 |  |  | 0.07 | 0.18 | 0.11 | 0.07 | 0.10 |
|  | TOP10 | 0.10 | 0.09 | 0.13 | 0.26 |  |  | 0.09 | 0.09 | 0.13 |  |  | 0.08 | 0.24 | 0.14 | 0.09 | 0.13 |
|  | TOP20 | 0.12 | 0.11 | 0.15 | 0.27 |  |  | 0.11 | 0.11 | 0.15 |  |  | 0.10 | 0.27 | 0.16 | 0.11 | 0.15 |
|  | TOP25 | 0.12 | 0.11 | 0.15 | 0.28 |  |  | 0.11 | 0.12 | 0.15 |  |  | 0.11 | 0.28 | 0.16 | 0.12 | 0.15 |
|  | TOP30 | 0.13 | 0.12 | 0.16 | 0.29 |  |  | 0.12 | 0.12 | 0.16 |  |  | 0.11 | 0.28 | 0.17 | 0.12 | 0.16 |
|  | TOP40 | 0.13 | 0.13 | 0.16 | 0.30 |  |  | 0.12 | 0.13 | 0.16 |  |  | 0.12 | 0.29 | 0.18 | 0.13 | 0.16 |
|  | TOP50 | 0.14 | 0.13 | 0.17 | 0.31 |  |  | 0.13 | 0.13 | 0.17 |  |  | 0.12 | 0.30 | 0.18 | 0.14 | 0.17 |
|  | TOP100 | 0.16 | 0.15 | 0.19 | 0.33 |  |  | 0.15 | 0.15 | 0.19 |  |  | 0.14 | 0.32 | 0.20 | 0.15 | 0.19 |
|  | TOP200 | 0.18 | 0.17 | 0.21 | 0.35 |  |  | 0.17 | 0.17 | 0.21 |  |  | 0.16 | 0.34 | 0.22 | 0.17 | 0.21 |
|  | TOP500 | 0.22 | 0.21 | 0.24 | 0.37 |  |  | 0.21 | 0.20 | 0.24 |  |  | 0.20 | 0.37 | 0.25 | 0.21 | 0.24 |
|  | TOP1000 | 0.26 | 0.25 | 0.28 | 0.37 |  |  | 0.26 | 0.24 | 0.28 |  |  | 0.24 | 0.41 | 0.29 | 0.25 | 0.28 |
| N05 (Tumor) | # of most common clones to get to 5% | 1.41 | 1.47 | 1.21 | 0.09 |  |  | 1.56 | 1.25 | 1.21 |  |  | 1.48 | 1.00 | 1.50 | 1.56 | 1.21 |
| N10 |  | 2.54 | 2.65 | 2.21 | 0.28 |  |  | 2.89 | 2.00 | 2.21 |  |  | 2.72 | 2.00 | 2.00 | 2.44 | 2.21 |
| N15 |  | 4.30 | 4.45 | 3.83 | 0.41 |  |  | 4.87 | 3.30 | 3.83 |  |  | 4.59 | 3.00 | 3.00 | 4.11 | 3.83 |
| N20 |  | 6.83 | 7.07 | 6.08 | 0.44 |  |  | 7.72 | 5.30 | 6.08 |  |  | 7.36 | 5.00 | 4.50 | 6.11 | 6.08 |
| N25 |  | 10.52 | 10.91 | 9.33 | 0.44 |  |  | 12.00 | 7.95 | 9.33 |  |  | 11.43 | 8.50 | 7.00 | 8.78 | 9.33 |
| N30 |  | 15.40 | 16.08 | 13.29 | 0.36 |  |  | 17.80 | 11.45 | 13.29 |  |  | 16.92 | 12.50 | 9.00 | 12.78 | 13.29 |
| N35 |  | 22.40 | 23.45 | 19.17 | 0.34 |  |  | 25.85 | 16.95 | 19.17 |  |  | 24.82 | 18.50 | 11.50 | 17.89 | 19.17 |
| N40 |  | 31.97 | 33.50 | 27.25 | 0.34 |  |  | 36.94 | 24.20 | 27.25 |  |  | 35.72 | 25.00 | 15.00 | 24.44 | 27.25 |
| N45 |  | 44.84 | 46.92 | 38.42 | 0.36 |  |  | 51.65 | 34.15 | 38.42 |  |  | 50.23 | 33.00 | 19.50 | 33.67 | 38.42 |
| N50 |  | 61.89 | 64.58 | 53.58 | 0.40 |  |  | 70.93 | 47.45 | 53.58 |  |  | 69.28 | 43.00 | 26.50 | 46.00 | 53.58 |
| N55 |  | 84.74 | 88.18 | 74.17 | 0.45 |  |  | 96.74 | 65.05 | 74.17 |  |  | 94.66 | 55.00 | 36.00 | 63.22 | 74.17 |
| N60 |  | 115.04 | 119.12 | 102.46 | 0.51 |  |  | 130.54 | 88.30 | 102.46 |  |  | 127.95 | 70.50 | 48.00 | 85.89 | 102.46 |
| N65 |  | 155.73 | 160.47 | 141.13 | 0.58 |  |  | 175.61 | 119.60 | 141.13 |  |  | 172.49 | 88.00 | 62.50 | 116.89 | 141.13 |
| N70 |  | 210.47 | 215.62 | 194.58 | 0.67 |  |  | 235.83 | 161.05 | 194.58 |  |  | 231.82 | 109.00 | 82.00 | 159.22 | 194.58 |
| N75 |  | 284.71 | 289.93 | 268.63 | 0.75 |  |  | 316.76 | 217.50 | 268.63 |  |  | 311.64 | 134.50 | 108.00 | 217.78 | 268.63 |
| N80 |  | 386.94 | 392.01 | 371.29 | 0.82 |  |  | 428.04 | 294.75 | 371.29 |  |  | 421.46 | 167.00 | 141.50 | 298.11 | 371.29 |
| N85 |  | 532.43 | 536.62 | 519.50 | 0.89 |  |  | 586.31 | 402.45 | 519.50 |  |  | 576.51 | 208.50 | 194.00 | 415.33 | 519.50 |
| N90 |  | 752.98 | 754.45 | 748.46 | 0.97 |  |  | 826.22 | 560.65 | 748.46 |  |  | 809.69 | 264.00 | 276.00 | 595.33 | 748.46 |
| N95 |  | 1133.38 | 1126.97 | 1153.13 | 0.92 |  |  | 1234.28 | 837.25 | 1153.13 |  |  | 1208.66 | 352.00 | 399.00 | 907.33 | 1153.13 |
| R05 | % of PBMC mapping to N05 clones | 0.04 | 0.04 | 0.03 | 0.29 |  |  | 0.05 | 0.02 | 0.03 |  |  | 0.04 | 0.00 | 0.13 | 0.03 | 0.03 |
| R10 |  | 0.05 | 0.06 | 0.03 | 0.09 |  |  | 0.07 | 0.03 | 0.03 |  |  | 0.06 | 0.11 | 0.13 | 0.04 | 0.03 |
| R15 |  | 0.07 | 0.07 | 0.09 | 0.53 |  |  | 0.08 | 0.04 | 0.09 |  |  | 0.07 | 0.11 | 0.13 | 0.06 | 0.09 |
| R20 |  | 0.11 | 0.10 | 0.13 | 0.46 |  |  | 0.10 | 0.10 | 0.13 |  |  | 0.11 | 0.11 | 0.13 | 0.06 | 0.13 |
| R25 |  | 0.14 | 0.13 | 0.18 | 0.36 |  |  | 0.14 | 0.11 | 0.18 |  |  | 0.14 | 0.23 | 0.19 | 0.07 | 0.18 |
| R30 |  | 0.18 | 0.17 | 0.20 | 0.50 |  |  | 0.17 | 0.17 | 0.20 |  |  | 0.18 | 0.25 | 0.19 | 0.11 | 0.20 |
| R35 |  | 0.22 | 0.22 | 0.24 | 0.60 |  |  | 0.21 | 0.23 | 0.24 |  |  | 0.22 | 0.25 | 0.25 | 0.18 | 0.24 |
| R40 |  | 0.25 | 0.25 | 0.26 | 0.81 |  |  | 0.24 | 0.27 | 0.26 |  |  | 0.25 | 0.26 | 0.31 | 0.21 | 0.26 |
| R45 |  | 0.29 | 0.29 | 0.28 | 0.97 |  |  | 0.28 | 0.31 | 0.28 |  |  | 0.29 | 0.38 | 0.37 | 0.22 | 0.28 |
| R50 |  | 0.32 | 0.32 | 0.33 | 0.89 |  |  | 0.31 | 0.34 | 0.33 |  |  | 0.32 | 0.39 | 0.47 | 0.26 | 0.33 |
| R55 |  | 0.36 | 0.36 | 0.36 | 0.91 |  |  | 0.35 | 0.38 | 0.36 |  |  | 0.36 | 0.49 | 0.52 | 0.30 | 0.36 |
| R60 |  | 0.40 | 0.41 | 0.39 | 0.72 |  |  | 0.40 | 0.42 | 0.39 |  |  | 0.40 | 0.55 | 0.59 | 0.38 | 0.39 |
| R65 |  | 0.44 | 0.45 | 0.42 | 0.57 |  |  | 0.44 | 0.46 | 0.42 |  |  | 0.44 | 0.56 | 0.61 | 0.42 | 0.42 |
| R70 |  | 0.48 | 0.49 | 0.45 | 0.48 |  |  | 0.48 | 0.51 | 0.45 |  |  | 0.48 | 0.61 | 0.64 | 0.47 | 0.45 |
| R75 |  | 0.53 | 0.53 | 0.50 | 0.52 |  |  | 0.53 | 0.55 | 0.50 |  |  | 0.53 | 0.62 | 0.67 | 0.52 | 0.50 |
| R80 |  | 0.57 | 0.58 | 0.55 | 0.48 |  |  | 0.57 | 0.59 | 0.55 |  |  | 0.57 | 0.66 | 0.72 | 0.55 | 0.55 |
| R85 |  | 0.63 | 0.63 | 0.60 | 0.47 |  |  | 0.63 | 0.65 | 0.60 |  |  | 0.63 | 0.69 | 0.78 | 0.60 | 0.60 |
| R90 |  | 0.69 | 0.70 | 0.67 | 0.43 |  |  | 0.70 | 0.70 | 0.67 |  |  | 0.70 | 0.71 | 0.81 | 0.66 | 0.67 |
| R95 |  | 0.78 | 0.79 | 0.77 | 0.47 |  |  | 0.79 | 0.78 | 0.77 |  |  | 0.79 | 0.77 | 0.85 | 0.75 | 0.77 |
| Sharing | # Clones, PBMC & Tumor | 2484.80 | 2453.22 | 2582.17 | 0.79 |  |  | 2609.37 | 2031.60 | 2582.17 |  |  | 2574.08 | 940.50 | 762.00 | 2346.00 | 2582.17 |
|  | % shared vs Tumor | 0.53 | 0.53 | 0.53 | 0.93 |  |  | 0.52 | 0.54 | 0.53 |  |  | 0.54 | 0.55 | 0.61 | 0.45 | 0.53 |
|  | % shared vs PBMC | 0.20 | 0.19 | 0.23 | 0.24 |  |  | 0.19 | 0.17 | 0.23 |  |  | 0.19 | 0.31 | 0.20 | 0.18 | 0.23 |
